# Host and *Helicobacter pylori* HtrA protease variants converge on Wnt/β-catenin signaling to drive stomach adenocarcinoma

**DOI:** 10.1080/19490976.2026.2704244

**Published:** 2026-07-23

**Authors:** Bodo Linz, Saiswaroop Rajaratnam, Nicole Tegtmeyer, Aakash Chhetri, Krishnasalini Gunanathan, Subbarao Kanchi, Suneesh Kumar Pachathundikandi, Venketesh Sivaramakrishnan, Steffen Backert

**Affiliations:** a Department of Biology, Division of Microbiology, Friedrich-Alexander Universität Erlangen-Nürnberg, Erlangen, Germany; b Disease Biology Lab, Department of Biosciences, Sri Sathya Sai Institute of Higher Learning, Anantapur, Andhra Pradesh, India; c Department of Physics, Sri Sathya Sai Institute of Higher Learning, Andhra Pradesh, Anantapur, India; d Department of Microbiology, School of Earth and Environmental Sciences, Babasaheb Bhimrao Ambedkar University, Lucknow, India

**Keywords:** β-catenin, CagA, E-cadherin, fibronectin, *Helicobacter pylori*, HtrA proteases, inflammation, interleukin-8, NF-κB, Wnt

## Abstract

*Helicobacter pylori-*associated stomach adenocarcinoma (STAD) represents a highly severe malady, with up to 1 million new cases annually. Here, we examined novel human and bacterial risk determinants as well as related signal transduction events associated with STAD development. *H. pylori* serine protease HtrA cleaves the junctional protein E-cadherin, which results in the disruption of epithelial cell connections, the release and nuclear accumulation of β-catenin, and the onset of epithelial-mesenchymal transition (EMT), a hallmark of many tumors. In addition, the injection of *H. pylori* oncoprotein CagA into host epithelial cells targets β-catenin-mediated cell proliferation and other cancer signaling pathways. By analyzing over 2,000 *H. pylori* genomes, we identified single-nucleotide polymorphism (SNP) variants of HtrA and CagA that are associated with STAD progression. In addition, we investigated the role of the human serine proteases HTRA1, HTRA2, HTRA3, and HTRA4 in STAD progression and linked the genetic and expression data with specific signaling pathways. Elevated HTRA1, HTRA2, and HTRA3 expression in STAD patients correlated with upregulated extracellular matrix (ECM) receptor interactions and signaling that are critical for EMT. Moreover, *H. pylori-*positive STAD patients exhibited increased epithelial cell signaling, chronic inflammation, transcription factor Wnt/β-catenin signaling, ECM damage and metastasis, and single-cell analyses showed a strong association between HTRA1, the receptor Wnt, β-catenin, and oncogene MYC expression. Analyses of mutations in human HTRA1 and *H. pylori* HtrA revealed a role in the up- or downregulation of STAD progression. Together, our data show that SNPs in human and *H. pylori* serine protease HtrA and CagA modulate cancer signaling in complex Wnt-/β-catenin and ECM signaling networks, and that the protein variants can be causative or protective factors. A signaling model is proposed that highlights the complex interplay of human and bacterial factors in critical tumor signaling events, which could serve as predictive STAD biomarkers in patients.

## Introduction

Cancer is still one of the most challenging health problems for humans worldwide. The Cancer Genome Atlas (TCGA) is a pioneering cancer genomics initiative that comprises 33 human cancer types, describing more than 20,000 primary cancer samples with corresponding controls (https://www.cancer.gov/ccg/research/genome-sequencing/tcga). This joint initiative of the National Cancer Institute (NCI) and the National Human Genome Research Institute (NHGRI) in the United States started in 2006 and produced various petabytes of cancer genomic, epigenomic, transcriptomic, and proteomic datasets. These datasets are still key for the diagnosis, prevention, and treatment of millions of human cancer patients.^1^ Gastric cancer (GC) is one of the most common fatal cancers, for which there are only limited treatment options.^2^ ^,^ ^3^ At the global scale, GC represents the fifth most widespread malignant cancer and the fourth leading cause of cancer-associated death.^4^ The GC prevalence rates depend on the geographical area and are particularly high in various countries of East Europe, Asia, and the Andean region of South America.^5^ According to the currently announced worldwide GLOBOCAN statistics for the year 2022, 968,784 new GC cases and 660,175 GC-related deaths were reported.^6^ In the United States, 30,300 new GC cases and 10,780 deaths were estimated for the year 2025.^7^ This disease is more common in men than in women, and the average age at diagnosis is approximately 68 years. The TCGA classified GC into four individual subtypes according to specific genetic and epigenetic features: (1) Epstein‒Barr virus-related GC, (2) GC with chromosomal instability, (3) GC with microsatellite instability-high, and (4) GC with genomic stability. The majority of GC cases (>90%) represent stomach adenocarcinomas (STAD).^8^ Recent advances in next-generation high-throughput sequencing technologies have substantially improved our understanding of cancer genetics at the molecular level. For example, a comprehensive recent study investigated pathogenic germline variants in 27 cancer-associated genes in several thousand samples from GC and control patients in Japan.^9^ Genetic investigation of this patient material has shown that GC development is significantly associated with 9 pathogenic germline variants, most notably in the cell junction gene encoding E-cadherin as well as some important genes controlling homologous recombination and DNA repair.^9^ These novel findings suggest that disturbance of the gastric epithelial barrier and impaired DNA repair processes could play a major role in GC progression.

Another important GC risk factor is the persistent bacterium *Helicobacter pylori*. This pathogen colonizes the stomach of approximately 50% of the human world population and is in competition with the gastric microbiota. Clinical and other studies highlighted a significant deviation in the composition of the gastric microbiota when *H. pylori* is present in persons with gastritis compared to patients with GC, implying that the microbiota may synergistically affect GC development, which is initially triggered by the pathogen.^3^ ^,^ ^5^ ^,^ ^10^ ^,^ ^11^ *H. pylori* encodes a variety of virulence factors, which hijack imperative pro-inflammatory and proliferative signaling pathways in the host, and trigger DNA damage.^5^ ^,^ ^12-14^ These signal cascades are primarily affected by the bacterial type IV secretion system (T4SS) in the *cag* pathogenicity island (*cag*PAI).^14^ In fact, this T4SS facilitates the delivery of effector molecules, such as the oncoprotein CagA and ADP-glycero-*β*-D-manno-heptose (ADP-heptose).^15^ While ADP-heptose profoundly activates transcription factor NF-κB-induced pro-inflammatory responses,^16-20^ injected CagA targets cell proliferation and anti-apoptotic signaling,^21-23^ both of which represent hallmarks of GC progression. A number of other *H. pylori* genes were also reported to affect GC outcome upon infection. These factors comprise the outer membrane adhesins BabA, SabA, HopH, HopQ, HopZ, AlpA/B, and others, which establish intimate bacteria‒host cell contact,^24-28^ as well as the VacA toxin allele *s1m1* that triggers intense cell vacuole formation and hampers T cell proliferation.^29^ ^,^ ^30^ Additionally, GC-linked activities include the induction of reactive nitrogen and oxygen species (RNS and ROS), inflammasome manipulation, and the introduction of DNA double-strand breaks (DSBs) in the host chromosome, resulting in chromosomal instability.^23^ ^,^ ^31-33^ In the same scenario, *H. pylori* secrete their serine protease HtrA that cleaves the junctional proteins E-cadherin, claudin-8 and occludin, which leads to the disruption of tight and adherens cell-to-cell junctions in the gastric epithelium, and thus, compromises the epithelial barrier.^34^ Cleavage of E-cadherin releases cytosolic β-catenin from the E-cadherin complex, followed by its translocation into the nucleus, where β-catenin triggers proliferative gene transcription.^23^ The disrupted cell connections allow transmigration of *H. pylori* to the basolateral side between the cells, where *H. pylori* employs the *cag*PAI-encoded T4SS to inject ADP-heptose and CagA into the epithelial cells.^34^ In addition to HtrA, intracellular CagA also targets E-cadherin and stimulates the release of β-catenin from the E-cadherin complex and its subsequent translocation into the nucleus and the onset of cell proliferation,^21^ ^,^ ^35^ ^,^ ^36^ which has been shown to be crucial for GC development in the Mongolian gerbil model system.^21^ Intracellular CagA is phosphorylated by the host kinases Src and Abl at the tyrosine residues of the C-terminal Glu-Pro-Ile-Tyr-Ala (EPIYA) motifs. There are four distinct EPIYA motifs, EPIYA-A, EPIYA-B, EPIYA-C, and EPIYA-D, all of which can be phosphorylated. Most *cagA*-positive strains contain three motifs: EPIYA-ABD in strains from East Asia (i.e., East Asian strains) and EPIYA-ABC in strains from other parts of the world, commonly known as Western strains.^37-39^ CagA with phosphorylated EPIYAs elicits the release and transfer of β-catenin into the nucleus, where it triggers cell proliferation and the development of adenocarcinoma. However, a study of 364 *H. pylori* strains with known gastrointestinal disease status, of which 171 were from patients with gastritis and 46 from GC patients, showed that CagA with the EPIYA-B amino acid allelic variant EPIYT was significantly less associated with GC compared to CagA with the EPIYA variant.^36^ The threonine residue of phosphorylated EPIYT forms a side chain hydrogen bond with an asparagine residue of phosphatidylinositol-3 (PI3) kinase, which in turn phosphorylates, and thus, activates the serine/threonine kinase AKT. Activated AKT then blocks NF-κB-mediated IL-8 transcription and inhibits glycogen synthase kinase-3β (GSK3), which results in reduced phosphorylation, and consequently, the stabilization of β-catenin complexes. In contrast, the alanine residue of the phosphorylated EPIYA fails to bind to PI3-kinase, and hence does not antagonize, but stimulates the transfer of β-catenin into the nucleus. Consequently, this A/T amino acid polymorphism in the EPIYA-B motif influences the outcome of gastric disease.^36^ ^,^ ^40^

While *H. pylori* commonly carries a single *htrA* gene copy in its genome,^41^ ^,^ ^42^ humans express four HTRA homologs, called HTRA1, HTRA2, HTRA3, and HTRA4.^43^ These HTRAs were described to play a role in numerous human cancers, such as breast, ovarian, endometrial and prostate cancer.^44-46^ However, almost nothing is known about the potential role of human HTRAs in GC development. In this study, we explored the roles of HTRA1, HTRA2, HTRA3, and HTRA4, and *H. pylori* CagA and HtrA in GC development. We hypothesize that human HTRA and at least two *H. pylori* proteins contribute to GC development and that all these proteins affect the same cell signaling pathways, particularly the Wnt/β-catenin cascade. In addition, we assessed the role of single nucleotide polymorphisms (SNPs) in the corresponding genes in GC development. These studies shed new light on the importance of human and *H. pylori* HtrA proteases and oncoprotein CagA in a complex Wnt-/β-catenin and ECM signaling network during STAD progression.

## Results

### Association of the *H. pylori* CagA EPIYA-B motif with gastric disease

**Figure 1.**
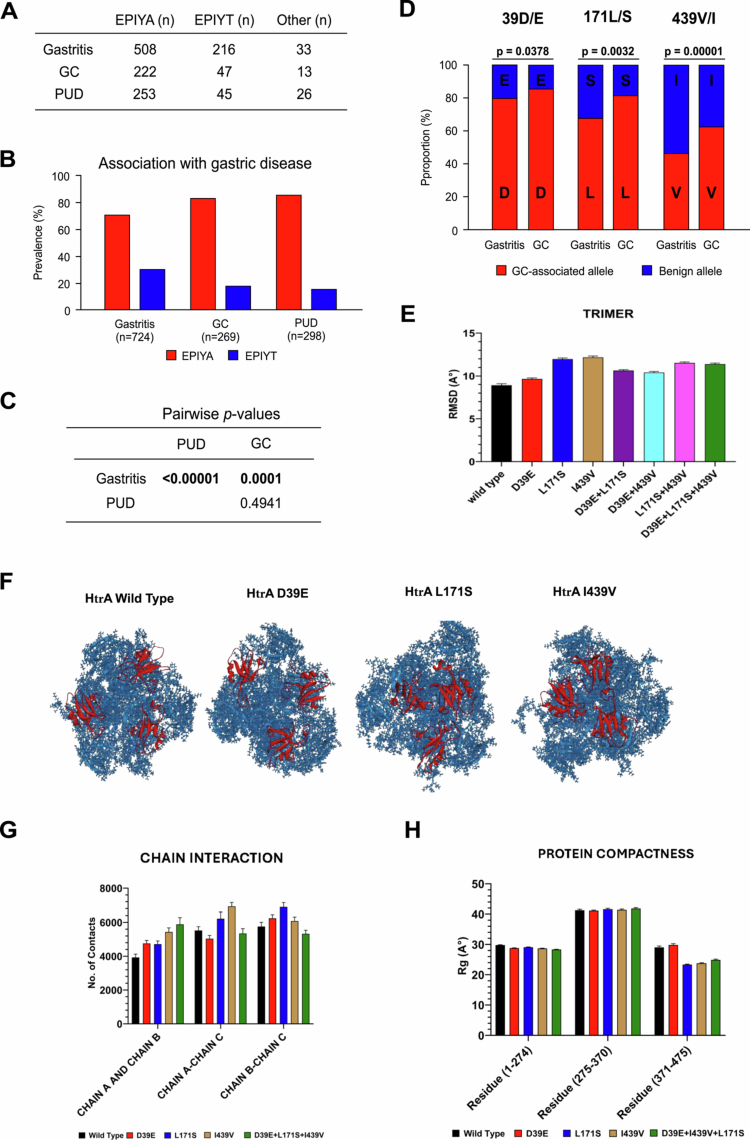
Association of the *H. pylori* CagA EPIYA-B motif and polymorphisms in bacterial HtrA with gastric disease. (A, B) Frequency of the EPIYA-B motif alleles Glu-Pro-Ile-Tyr-Ala (EPIYA) and Glu-Pro-Ile-Tyr-Thr (EPIYT) in *H. pylori* genomes from patients with gastric disease. (C) The two-tailed *p*-value of Fisher’s exact test of EPIYA vs. EPIYT frequency shows significant correlation with gastric disease. (D) Significant association of amino acid polymorphisms 39D/E, 171L/S, and 439V/I in *H. pylori* serine protease HtrA with the development of gastric cancer. (E) Bar graph representing Root Mean Square Deviation (RMSD) profiles of *H. pylori* wild-type and mutant HtrA trimers during molecular dynamics simulation (after 300  ns). (F) Structural visualization of the wild-type and D39E, L171S, and I439V mutant HtrA proteins after 300 ns molecular dynamics simulation, showing noticeable conformational alterations of the HtrA trimers (blue), particularly in the PDZ domain (red). (G) Averaged number of close contacts between Chains A, B, and C of HtrA wild-type and mutant proteins (last 50  ns). (H) Averaged Rg (radius of gyration) of HtrA in wild-type and D39E, L171S, I439V, and D39E + L171S + I439V mutants (last 50 ns).

STAD represents one of the most widespread forms of GC, which gradually developed over many years or decades. Because of the clear lack of early symptoms, many patients are diagnosed at an advanced stage, making it very difficult to treat them effectively. We compiled and analyzed a dataset of 2,267 *H. pylori* isolates of worldwide origin with known disease status (see Materials and Methods) for the presence of the C-terminal EPIYA motifs of the *H. pylori* oncoprotein CagA. Of particular interest was the EPIYA-B motif that was previously shown to affect the disease outcome in a dataset of 364 *H. pylori* strains.^36^ A tblastn search revealed that 1,736 genomes contained the CagA EPIYA-B motif, of which 1,650 contained the EPIYA (1,229 genomes) or EPIYT (421 genomes) allele variants, and 86 genomes contained other variants (see Methods). Among the 1,736 genomes, 1,363 genomes were from patients with non-atrophic gastritis (NAG, *n* = 757), gastric cancer (GC, *n* = 282), and peptic ulcer disease (PUD, *n* = 324), which were analyzed further. A total of 983 genomes contained the amino acid variant Glu-Pro-Ile-Tyr-Ala (EPIYA), 308 genomes contained the Glu-Pro-Ile-Tyr-Thr (EPIYT), and 72 genomes contained other variants, such as ESIYA, ESIYT, EPVYT, EPVYA, and others (Figure 1A). The frequency of the EPIYA-B amino acid variants EPIYA and EPIYT was significantly associated with gastric disease (Figure 1A–C). The EPIYA variant was significantly more frequent in isolates from patients with GC and PUD compared to patients with NAG (Fisher’s exact test: *p* = 0.0001 and *p* = <0.00001, respectively), showing EPIYA-to-EPIYT ratios of 70:30 for NAG isolates, 83:17 for GC isolates, and 85:15 for PUD isolates. Overall, our data confirmed and expanded on the data from a much smaller previously published dataset.^36^

### *H. pylori* HtrA variants that energetically stabilize the protease are associated with increased stomach cancer

An analysis of amino acid polymorphisms in the *H. pylori* serine protease HtrA of the same genomes (757 gastritis; 282 GC) revealed disease association for a glutamic acid variant (39D) in a glutamic acid/aspartic acid polymorphism at amino acid position 39 (polymorphism 39D/E), a leucine variant (171L) at a leucine/serine polymorphism in the protease domain of the protein at position 171 (polymorphism 171L/S), and a valine variant (439 V) at a valine/isoleucine polymorphism at position 439 (polymorphism 439V/I) (Figure 1D). HtrA allele variants 39D, 171L, and 439V were significantly associated with GC compared to gastritis (Fisher's exact test: *p* = 0.0378, *p* = 0.0032, *p* = 0.00001, respectively). Molecular dynamics (MD) simulations of the wild-type (HtrA allele variants 39D, 171L, and 439I), single, and a combination of double and triple mutants were performed to understand the role of the amino acid changes in STAD progression. The analysis, performed after the protein structures stabilized (300 ns) in the simulated environment, revealed that the individual changes affected the secondary protein structure and the stability of the protease trimer, which is the primary proteolytic form. The D39E change showed a more stabilizing effect, as shown by the largely unaffected Root Mean Square Deviation (RMSD) (Figure 1E, Supplementary Figure 1B). In contrast, L171S and I439V changes, as well as a combination of double and triple mutants, energetically destabilized the HtrA trimer structure, and thus, resulted in weaker HtrA variants that displayed considerably larger RMSD (Figures 1E, F, Supplementary Figure 1) and Root Mean Square Fluctuations (RMSF) per residue compared to the 39D-171L-439I-HtrA wild-type (Supplementary Figure 2). The visualization of the MD simulations showed that the mutations affected the overall conformation of the HtrA trimers in comparison to the HtrA wild-type trimers (Figure 1F). In particular, there were noticeable conformational alterations in the PDZ domain (red ribbons). *H. pylori* with 171L HtrA were previously shown to inflict severe epithelial damage through proteolytic cleavage of cell junction proteins, which triggered the Wnt/β-catenin pathway, releasing β-catenin from the E-cadherin-β-catenin complex and its accumulation in the nucleus.^23^ In addition, the destroyed cell junctions allows *H. pylori* migration to the basolateral side of the epithelial cells, where the bacteria inject CagA and ADP-heptose into the host cells, which enhances β-catenin-mediated signaling and induces NF-κB-mediated inflammation as well as DSBs in the host.^23^ In contrast, the L171S mutation was shown to destabilize the HtrA trimers,^23^ ^,^ ^47^ confirming the modeling results. This led to reduced proteolytic HtrA activity and, consequently, markedly reduced epithelial damage as well as an attenuation of β-catenin-mediated effects.^23^ Thus, changes in HtrA stability and activity caused by the protein variants identified in the GWAS correlate with the degradation of E-cadherin and β-catenin-mediated Wnt signaling. Accordingly, isolates from STAD patients showed a higher ratio of malignant versus benign HtrA alleles compared to isolates from patients with gastritis (Figure 1D), validating *H. pylori* HtrA as a STAD risk factor and providing mechanistic insights into its association with GC.

The PDZ domain in the protein was found to affect the conformational state of HtrA trimers in the mutants. MD structural visualization identified conformational changes in the PDZ domain of the mutants (Figure 1F), and chain interaction studies of close contacts between atoms in the trimeric state showed enhanced chain contacts in the mutant compared to wild-type HtrA (Figure 1G). Protein compactness, which was assessed using the radius of gyration (Rg), showed increased packing of the PDZ2 domain in the L171S and I439V mutants (Figure 1H), which can lead to reduced atomic fluctuations. In agreement, an RMSF analysis of only amino acid residues 371–475 of the PDZ2 domain showed significant fluctuations in the wild type, whereas the fluctuations were reduced in the mutants (not shown).

### Origin and distribution of *H. pylori* HtrA and CagA EPIYA-B variants

**Figure 2.**
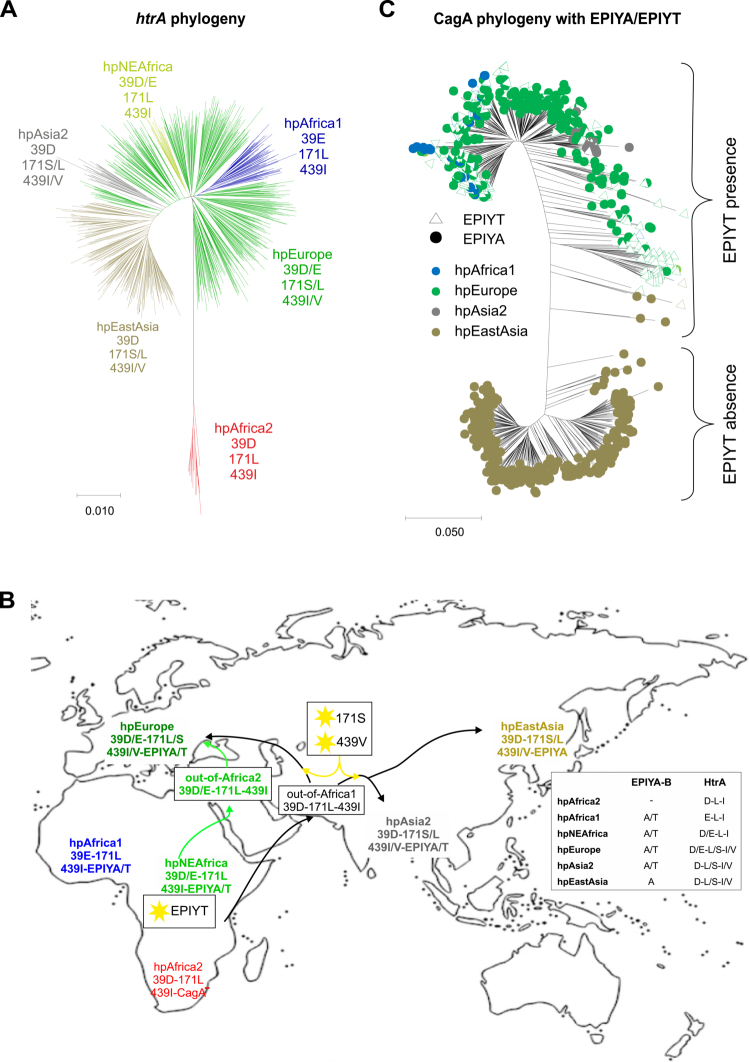
Evolution of SNPs in *H. pylori* serine protease HtrA and its association with gastric disease. (A) Neighbor-joining tree of the *htrA* sequences with the distribution of corresponding alleles in the biogeographic populations of *H. pylori*. (B) Neighbor-joining tree showing the CagA phylogeny and distribution of EPIYA-B EPIYA (filled circle) and EPIYT (open triangle) alleles. EPIYT is absent in CagA from *H. pylori* from East Asia. (C) Origin and biogeographic spread of the HtrA alleles and CagA EPIYA-B alleles. The HtrA alleles 39D, 39E, 171L, and 439I were the ancestral alleles in Africa and were spread globally after the migrations of modern humans out of Africa. Alleles 171S and 439V subsequently originated in Asia and were distributed with human migrations across Asia and Europe. The ancestral CagA EPIYA allele of the EPIYA-B motif was supplemented with the attenuated EPIYT allele before the out-of-Africa events.

The *htrA* gene is conserved and present in all isolates regardless of the geographic origin.^23^ ^,^ ^39^ ^,^ ^42^ However, the individual HtrA alleles are variably present among the biogeographic populations. All the genomes of the three African *H. pylori* populations hpAfrica1, hpAfrica2, and hpNEAfrica contained the 171L and 439I alleles. HpAfrica2 isolates carry the 39D variant, and hpAfrica1 isolates carry the 39E variant, whereas both 39D and 39E are found among hpNEAfrica isolates (Figure 2A). In contrast, 171L and 171S as well as 439I and 439V are present among isolates from the other populations, hpEurope, hpAsia2, and hpEastAsia. The genomes of the two Asian populations contained only 39D but not 39E. The biogeographic distribution of the alleles suggests the spread of the ancestral 39D-171L-439I HtrA variant out-of-Africa, after which the 171S and 439V alleles originated in Asia before the divergence of hpEastAsia from the hpAsia2 population (Figure 2B). A second out-of-Africa event ^48^ resulted in the spread of *H. pylori* with 39E HtrA from northeast Africa into the Near East, where the African isolates hybridized with *H. pylori* of the local hpAsia2, which gave rise to hpEurope that is known to be a hybrid population.^49^ ^,^ ^50^

In contrast to the ubiquitous *htrA*, the *cagA* gene is only variably present among the *H. pylori* isolates. It is present in all hpEastAsia, hpAsia2, and hpAfrica1 isolates, is variably present among isolates of the hpEurope and hpNEAfrica populations, but is absent from the population hpAfrica2.^39^ ^,^ ^51^ ^,^ ^52^ A neighbor-joining tree of the CagA protein showed both EPIYA and EPIYT alleles in the hpEurope, hpAfrica1, and hpAsia2 isolates, but the absence of the EPIYT variant from hpEastAsia isolates (Figure 2C). Analysis of additional genomes without disease information revealed both EPIYA and EPIYT among hpNEAfrica isolates (Linz and Backert, unpublished data). The presence of EPIYT in Africa suggests that this variant originated before the out-of-Africa migrations (Figure 2B).

### Expression of human serine proteases HTRA1 and HTRA3 is elevated in stomach adenocarcinoma

**Figure 3.**
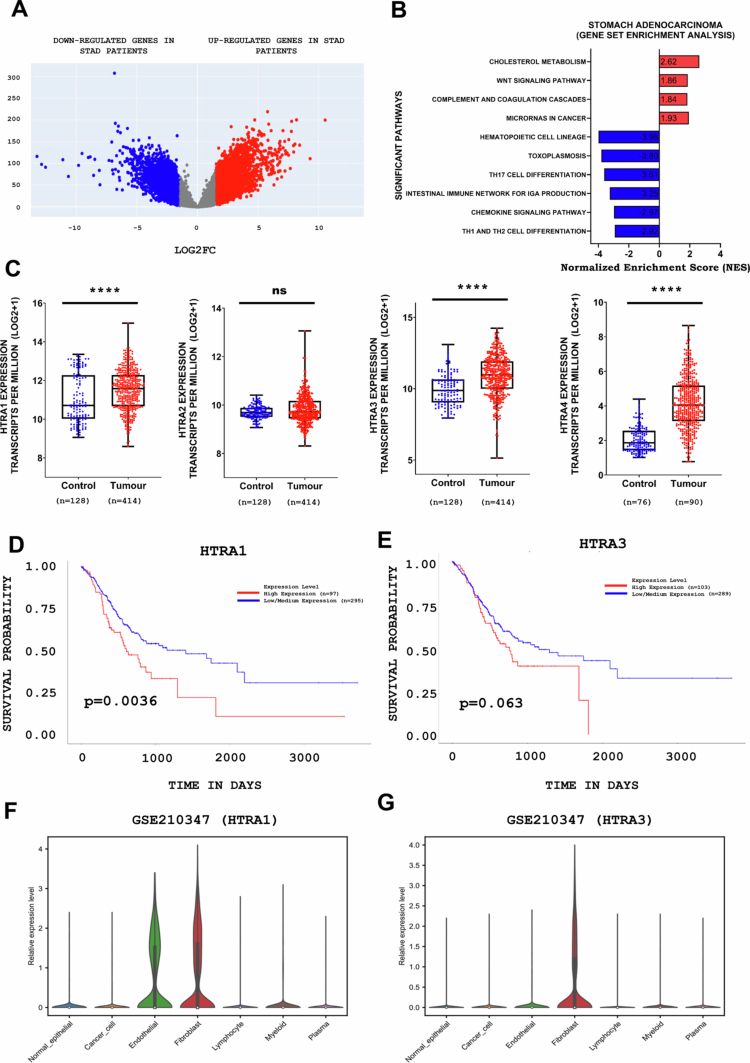
Human HTRA serine peptidase expression is elevated in stomach adenocarcinoma (STAD) patients. (A) Volcano plot representing differentially up-regulated and down-regulated genes in TCGA STAD datasets. Red color represents up-regulated and blue color represents down-regulated genes. The figure was generated using UCSC Xena (xena.ucsc.edu). (B) Bar graph representing significant pathways obtained from Gene Set Enrichment Analysis (GSEA) of STAD patients compared to benign tissues [TCGA gdac.broadinstitute.org, (*n* = 415, tumors), (*n* = 34, benign) FDR < 0.05]. (C) Box plots showing elevated HTRA transcript levels in primary tissues of STAD samples compared to benign tissue in the TCGA dataset (****P < 0.0001, *P < 0.05, Mann‒Whitney U test). (D) Survival curve representing a significantly higher death rate in STAD patients with increased HTRA1 expression [High HTRA1 (*n* = 97), low/medium HTRA1 (*n* = 295), *p* < 0.05, Wilcoxon test)]. (E) Survival curve representing a significantly higher death rate in STAD patients with increased HTRA1 expression [High HTRA3 (*n* = 103), low/medium human HtrA3 (*n* = 289), *p* < 0.05, Wilcoxon test]. (F, G) Violin plot representing the HTRA1 and HTRA3 expression levels in different cell types obtained from the STAD single-cell dataset (GSE210347). The UMAP and cell numbers are provided in Supplementary Figure 5A and B. The figures were generated using Single Cell Cancer Explorer (bianlab.cn/scCancerExplorer/home).

Phylogenetic analyses showed that homologs of HtrA serine proteases are present in bacteria, archaea, and eukaryotes, which suggests an evolutionarily conserved HtrA protein structure and functions among cellular organisms, including *H. pylori* HtrA and human HTRAs (Supplementary Figure 3). Human HTRA serine proteases are frequently found to be upregulated in various human cancer tissues and other diseases.^44^ ^,^ ^46^ However, their putative relevance in STAD development is yet unclear. To investigate this in detail, we identified differentially expressed genes and performed Gene Set Enrichment Analysis (GSEA) of TCGA STAD samples (tumor: *n* = 415; normal: *n* = 34) using an FDR cutoff of 0.05 (Figure 3A). STAD patients showed a strong positive enrichment for pathways associated with cholesterol metabolism, Wnt signaling, complement and coagulation cascades, and microRNA activity in cancer, while pathways related to hematopoietic cell lineage, toxoplasmosis, differentiation of Th1/Th2/Th17 cells, intestinal immune regulation, and chemokine signaling were negatively enriched (Figure 3B). These results align well with known functions of HTRA proteases, as secreted HTRA1 is known to degrade the ECM, to affect canonical Wnt signaling, and to interact with coagulation and fibrinolytic processes.^44^ The expression of HTRA2, another family member, is frequently associated with cancer phenotypes shaped by miRNA regulation.

Further, we measured the HTRA1, HTRA2, HTRA3, and HTRA4 expression in the TCGA STAD datasets (Figure 3C). HTRA1, HTRA3, and HTRA4 showed significantly higher levels in tumor tissues compared to controls. Classification of tumors into tumor grades showed significantly higher HTRA4 expression in Grade 3 tumors compared to Grade 2 tumors, while HTRA1 and HTRA3 showed a slightly increased expression in Grade 3 tumors (Supplementary Figure 4A-D). The critical role of HTRA in STAD progression was assessed in Kaplan–Meier plots. Survival curve analysis showed a significant association between high HTRA1 expression and reduced survival of STAD patients, while HTRA2, HTRA3, and HTRA4 expression were insignificant (Figure 3D, E, Supplementary Figure 4E, F). Further, single-cell datasets (GSE210347) with 232 samples (normal = 31; adjacent = 54; tumor = 148) from 164 donors were analyzed (Supplementary Figure 5A-B). Expression of HTRA1 and HTRA3 were found to be highly expressed in endothelial cells and fibroblasts (Figure 3F-G), while HTRA2 and HTRA4 did not show any significant difference (Supplementary Figure 5C-D). The single-cell results were then validated with a spatial transcriptomic Single Seq dataset (GSE167297) from five GC patients (Supplementary Figure 6A, B). Consistent with previous results, HTRA1 and HtrA3 were found to be highly expressed in endothelial cells and fibroblasts, while HTRA2 and HTRA4 did not show any significant difference (Supplementary Figure 6C–F). Taken together, our results show that human HTRA1 and HTRA3 are secretory proteins that might play a relevant role in tumor progression associated with STAD.

### High HTRA1 and HTRA3 expression in TCGA STAD patients showed enrichment of ECM receptor interaction and signaling pathways critical for EMT

**Figure 4.**
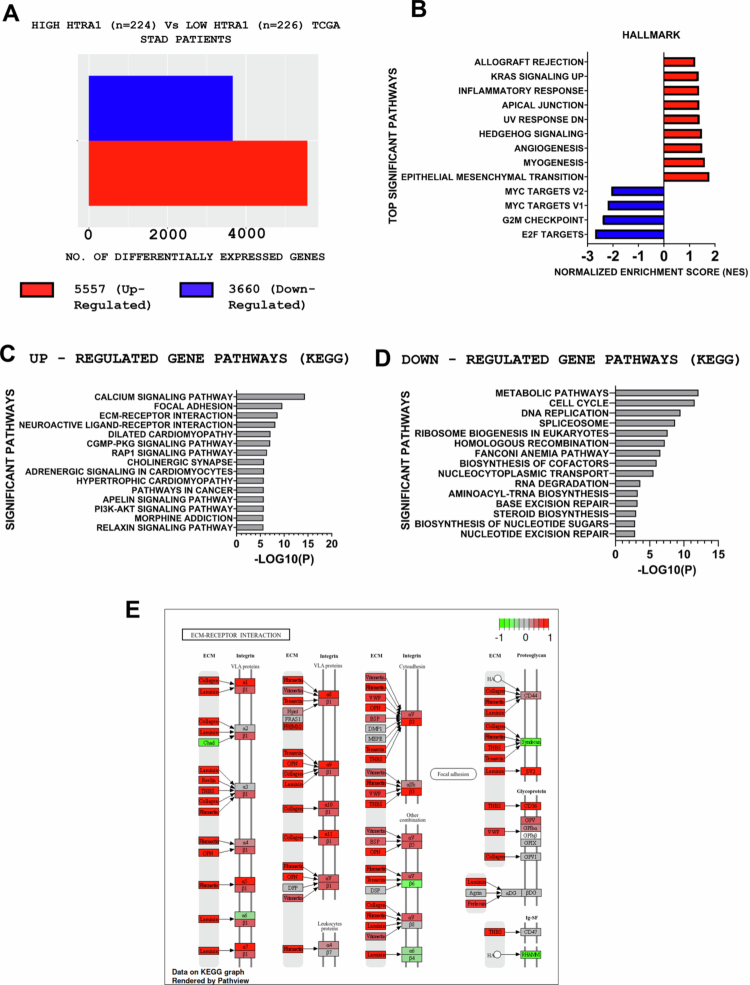
Enrichment analysis of STAD tumors with high and low human HTRA1 expression levels, using the KEGG and Hallmark databases, revealed a significant enrichment of ECM signaling pathways. (A) Bar graphs representing significant genes obtained from differential human gene expression between high- and low-HTRA1-expressing stomach adenocarcinoma (STAD) tumors from TCGA patients (high-HTRA1 patients (224), low-HTRA1 patients (226), adjusted FDR < 0.05, Log FC ± 1). (B) Significantly enriched pathways (FDR ≤ 0.05) identified by Gene Set Enrichment Analysis (GSEA) in high vs. low HtrA1 expression groups in STAD tumor. Data from the Hallmark Database. (C, D) Significantly enriched pathways (FDR ≤ 0.05) identified by Pathway Enrichment Analysis of high and low HTRA1 expression groups in STAD tumors. (E) Visualization of the Wnt signaling pathway (KEGG) overlaid with significantly expressed genes derived from differential expression analysis between the high and low HTRA1 expression groups in TCGA STAD samples. Down-regulated genes (−1) in green, up-regulated genes (1) in red.

To probe more into the role of HTRA1 in the disease progression of STAD, we grouped corresponding TCGA samples into high- (226) and low-expressing (224) patients. Differential gene expression between the groups identified 5,557 up-regulated and 3,660 down-regulated genes (FDR < 0.05, Log FC ± 1) (Figure 4A). To gain insight into the underlying biological processes and functional implications, the differentially expressed genes were subsequently mapped to biological pathways using Hallmark and KEGG databases. Gene Set Enrichment Analysis (GSEA) using Hallmark showed significant positive enrichment of signaling pathways comprising EMT, angiogenesis, KRAS signaling, inflammatory responses, and hedgehog genes, as well as negative enrichment of MYC and E2F targets, and G2M checkpoints (Figure 4B). Pathway enrichment of up-regulated genes using KEGG showed significant enrichment of genes involved in ECM-receptor interaction, PI3-kinase that activates serine/threonine protein kinase B (PKB, also called AKT) signaling, and other pathways. In contrast, down-regulated genes were associated with the cell cycle, DNA replication, and DNA repair mechanisms (Figure 4C, D). Compromised ECM components are associated with tumor invasion, migration, and metastasis. Inactivation of DNA repair mechanisms leads to increased mutation accumulation and genomic instability, and activated PI3K/AKT kinases result in enhanced cell survival and resistance to apoptosis of already compromised cells. We mapped the differentially expressed genes onto the KEGG pathway map and found that several upregulated genes were associated with ECM receptor signaling, including those related to the expression of fibronectin, collagen, tenascin, laminin, von Willebrand factor (VWF), bone sialoprotein (BSP), thrombospondin (THBS), and vitronectin, showing significant enrichment of the ECM-receptor interaction pathway (Figure 4E).

To validate the gene-set enrichment analysis at the protein level, we performed a kinase enrichment analysis of the significantly upregulated genes from a proteomic dataset (https://maayanlab.cloud/X2K/). The analysis showed enrichment of several kinases, including GSK3, which is part of the β-catenin destruction complex in the Wnt signaling pathway (Supplementary Figure 7). Since HTRA1 expression directly influences Wnt signaling,^53^ the proteomics dataset was compared with the datasets “high vs. low HTRA1 expression” and “*H. pylori*-infected vs. uninfected”. The analysis revealed common enrichment of several kinases, including GSK3, across all three datasets (Supplementary Figure 8), which confirmed the gene expression data at the protein level.

**Figure 5.**
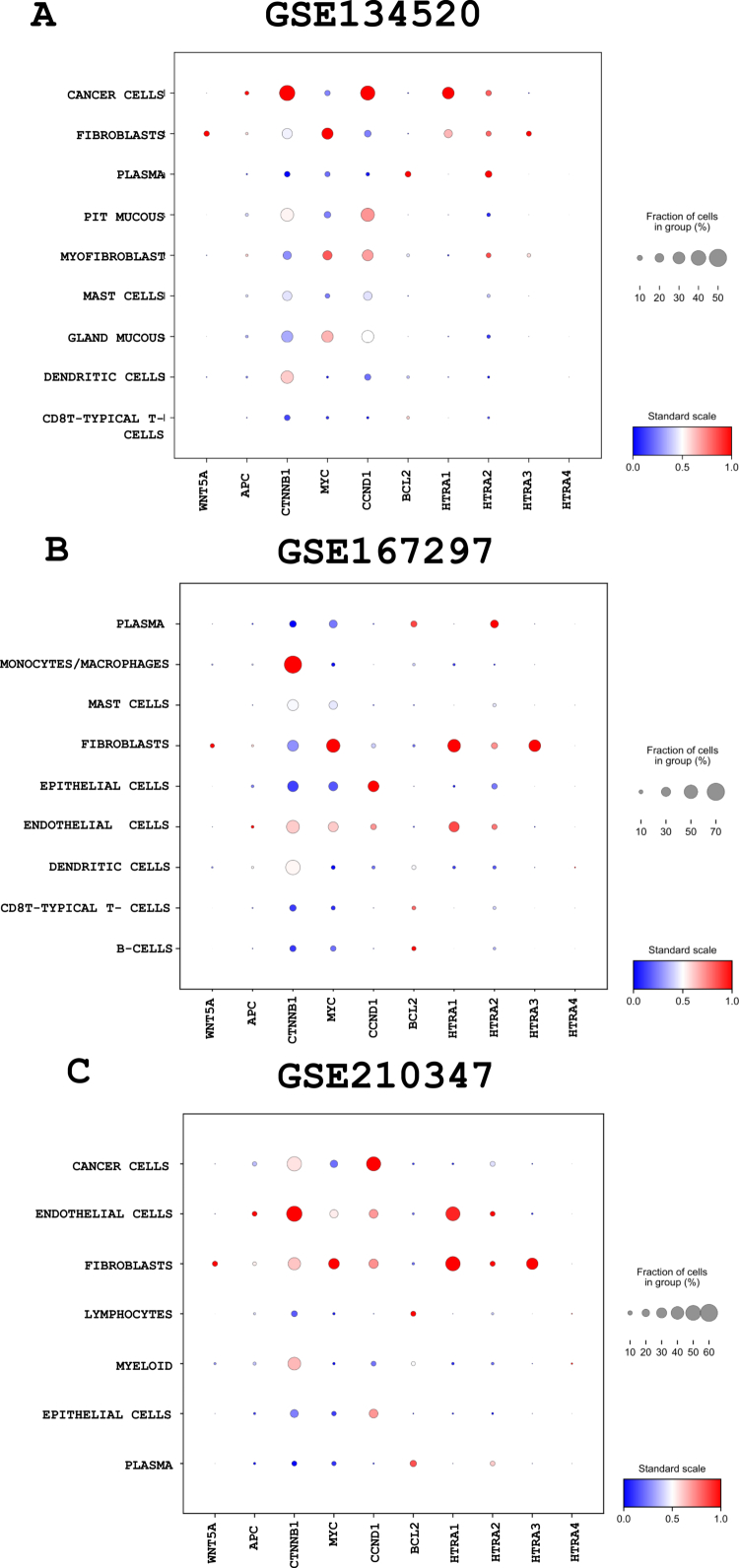
Single-cell analysis shows a strong association between Wnt signaling and human HTRA1 expression. (A) Dot plot representing the enrichment ratio of HTRA and Wnt signaling genes in different cell types in the STAD Single Seq dataset (GSE134520). The figure was generated using Single Cell Cancer Explorer (bianlab.cn/scCancerExplorer/home). (B) Dot plot representing the enrichment ratio of HTRA and Wnt signaling genes in different cell types in the STAD Single Seq dataset (GSE167297). The figure was generated using the Single Cell Cancer Explorer. (C) Dot plot representing the enrichment ratio of human HTRA and Wnt signaling genes in different cell types in the STAD single Seq dataset (GSE210347). The figure was generated using Single Cell Cancer Explorer.

Inflammatory response pathways were positively enriched in high HTRA1-expressing patients (Figure 4B). Although HTRA1 is not directly expressed in immune cell populations, elevated HTRA1 expression in fibroblasts, tumor cells, and endothelial cells (Figure 5) may influence the tumor microenvironment and promote inflammatory signaling. Therefore, we performed immune cell infiltration analyses to further characterize the corresponding immune landscape (Supplementary Figure 9). Assessment of immune cell infiltration in tumors of high (*n* = 100) versus low HTRA1 (*n* = 100) STAD patients using TIMER, EPIC, CIBERSORT, CIBERSORT-ABS and XCELL algorithms revealed significantly increased tumor infiltration by CD4+ T-cells, CD8+ T-cells, B-cells, macrophages, neutrophils, and mast cells, confirming the induction of inflammatory responses in high HTRA1 expressing patients (Supplementary Figure 9).

Differential gene expression analysis between High (*n* = 224) and Low (*n* = 226) HTRA3-expressing patients identified 5,215 up-regulated and 4,753 down-regulated genes (FDR < 0.05, Log FC ± 1) (Supplementary Figure 10A). Hallmark pathway analysis showed positive enrichment of hypoxia, inflammation signaling, and EMT and negative enrichment of MYC and E2F targets (Supplementary Figure 10B). KEGG pathway analysis of the significantly up-regulated genes revealed enrichment of pathways such as Cell Adhesion Molecules (CAMs), focal adhesion, and calcium signaling, as well as ECM-receptor interactions, whereas down-regulated genes were enriched for pathways associated with metabolism and the cell cycle (Supplementary Figure 10C, D). Similar to HTRA1, genes associated with ECM-receptor interactions were found to be up-regulated (Supplementary Figure 10E). We also performed similar analyses with HTRA2 and HTRA4 (Supplementary Figures 11 and 12). While HTRA2 showed downregulation of genes associated with ECM–receptor interactions, HTRA4 was found to be associated with upregulated ECM-receptor interactions. However, HTRA4 was poorly expressed in the samples. Overall, HTRA1 might play a more prominent role with increased ECM receptor interaction in endothelial cells, which could potentially lead to ECM remodeling and thereby facilitate tumor progression.

### Single-cell analysis reveals a strong association between HTRA1, Wnt, and *β*-Catenin expression and between HTRA1 and angiogenesis

To probe the cell-specific role of the Wnt cascade in STAD, we analyzed the expression of critical genes from the Wnt signaling pathway using a STAD single-cell sequencing dataset (GSE134520, GSE167297, and GSE210347). The results showed a positive correlation between HTRA1 and β-catenin (CTNNB1), and a consistent negative correlation was observed between MYC and β-catenin expression in fibroblasts (Figure 5A–C). Additionally, downstream transcription factors such as MYC showed weaker enrichment in endothelial cells compared to fibroblasts (Figure 5A–C). While the different data sets showed differences in HTRA1 and HTRA3 expression in cell types such as endothelial cells, cancer-associated fibroblasts, and stomach cancer cells, there was functional convergence in the Wnt signaling and cell cycle pathways, revealing a strong association between HTRA1 expression and Wnt signaling in STAD progression.

Both HTRA1 and β-catenin expression were high in the endothelial cells of STAD patients. Endothelial cells are known to be associated with ECM remodeling and VEGF–Wnt crosstalk, and HTRA1 expression was indeed found to stimulate endothelial tubulation and angiogenesis (Figure 4B). Single-cell analyses of samples from high HTRA1-expressing patients (GSE210347, GSE167297, and GSE134520) revealed elevated expression of angiogenesis-related markers, including Vascular Endothelial Growth Factor A (VEGFA), Fibroblast Growth Factor 1 (FGF1), Platelet-Derived Growth Factor Subunit A (PDGFA), and Angiopoietin-1 (ANGPT1) (Supplementary Figure 13). In addition, the expression of tubulation-related markers such as VE-cadherin (CDH5) and CD34 was also elevated, particularly in endothelial cells and in cancer cells (Supplementary Figure 13), validating enrichment of the angiogenesis pathway in high HTRA1-expressing patients (Figure 4B).

### *H. pylori-*positive STAD tumors show a strong association with increased epithelial cell signaling, chronic inflammation, Wnt/β-catenin signaling, ECM damage, and metastasis

**Figure 6.**
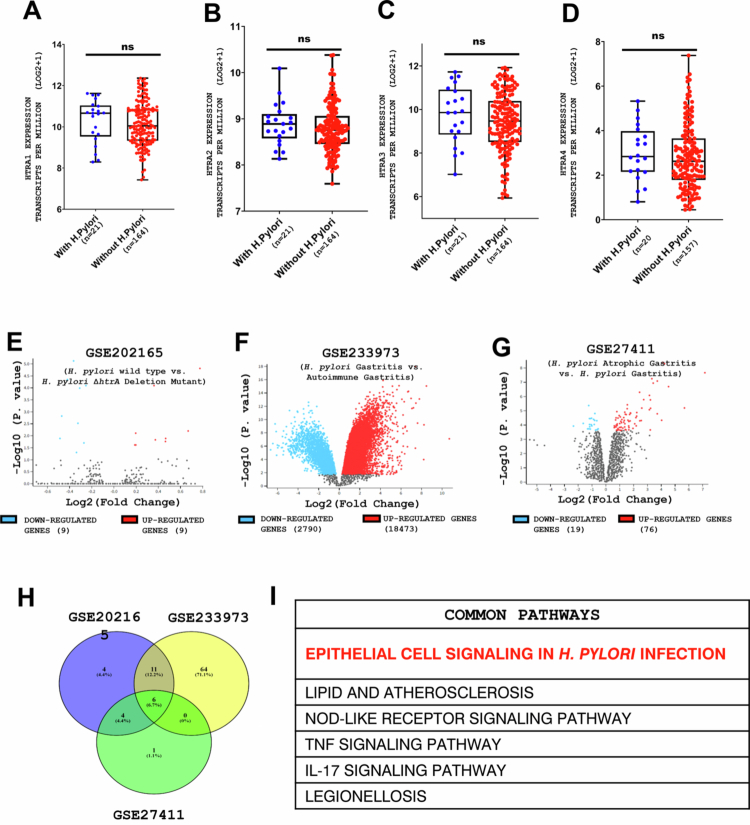
STAD tumors associated with *H. pylori* shows strong association with increased epithelial cell signaling, chronic inflammation, Wnt/β-catenin signaling, ECM damage, and metastasis. (A–D) Box plots of HtrA transcript levels in primary tissues of STAD tumors with and without *H. pylori* infection. (E–G) Volcano plots of differentially expressed genes obtained from the GEO datasets GSE202165 (*H. pylori* wild-type *vs*. *H. pylori* Δ*htrA* deletion mutant), GSE233973 (*H. pylori* gastritis *vs.* autoimmune gastritis), and GSE27411 (*H. pylori* atrophic gastritis *vs*. *H. pylori* non-atrophic gastritis) [adjusted P ≤ 0.05]. (H–I) Venn diagram and table representing common pathways obtained between the datasets [adjusted *P* ≤ 0.05].

Given that *H. pylori* HtrA is a risk factor associated with STAD progression (Figure 1), we further examined the transcriptomic profiles of *H. pylori*-infected and non-infected STAD patients. *H. pylori-*positive STAD patients showed slightly higher expression levels of HTRA1, HTRA2, HTRA3, and HTRA4, although these differences were not statistically significant (Figure 6A–D). These findings were further validated using independent gene expression datasets (GSE202165, GSE233973, and GSE27411) (Figure 6E–G). Pathway enrichment analysis of significant genes across these datasets identified six common pathways, including epithelial cell signaling during *H. pylori* infection (Figure 6H–I). *H. pylori* infection with CagA-positive strains is known to perturb gastric epithelial cell signaling, with a pronounced impact on the β-catenin pathway, thereby contributing to GC development.^21^ ^,^ ^36^

### Structural destabilization of HTRA1 by point mutations dampens cancer signaling and increases patient survival

**Figure 7.**
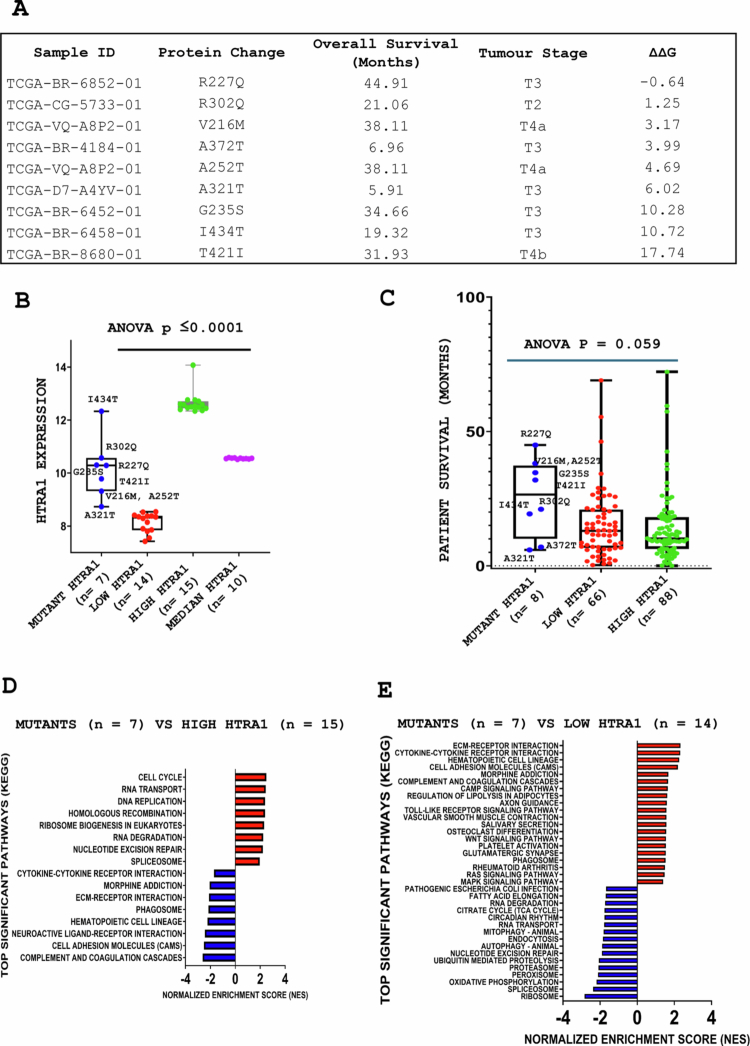
Human HTRA1 point mutants are associated with reduced STAD progression. (A) Table representing TCGA STAD patients with survival, tumor grade, and FoldX free energy calculation for different HTRA1 point mutants. HTRA1 mutations V216M and A252T were identified in the same patient. (B) Box plot representing the HTRA1 transcript levels of the HTRA1 mutant, HTRA1 low transcription, HTRA1 high transcription, and HTRA1 median transcription patient groups. (C) Box plot representing the survival of STAD patients with mutant HTRA1, low HTRA1 expression, and high HTRA1 expression. (D) Bar graphs summarizing significantly enriched pathways (FDR ≤ 0.05) identified by Gene Set Enrichment Analysis (GSEA) comparing mutant and high HTRA1 expression patient groups in STAD tumors, using the KEGG Database. (E) Bar graphs summarizing significantly enriched pathways (FDR ≤ 0.05) identified by Gene Set Enrichment Analysis (GSEA) comparing mutants and low HTRA1 expression patient groups in STAD tumors, using the KEGG Database.

Since HTRA1 expression showed a strong correlation with gastric disease progression, we sought to determine whether certain point mutations could confer a protective function by delaying disease progression. To this end, we identified HTRA1 point mutations from TCGA STAD datasets and computed free energy (ΔΔG) calculations (Figure 7A). Almost all point mutations exhibited positive ΔΔG values, which indicates significant structural perturbation and detrimental effects on protease activity. MD simulations of the protein structure showed significant changes in the RMSD values of the mutant compared to wild-type HTRA (Supplementary Figure 14A–C), which were accompanied by marked changes in secondary structures (Supplementary Figure 14D–F) and by a predicted decrease in HTRA stability, showing a mechanistic pathway for its association with STAD. Further, we asked if structural perturbations in HTRA1 could impact oncogenic signaling and influence patient survival independent of its expression level, as observed in Figure 7B. We examined differential expression sets in the transcriptome between mutants and non-mutants across patients with high, median, and low HTRA1 expression. Mutants showed negative enrichment of ECM receptor interaction compared with high-expressing patients, while positive enrichment was observed in low HTRA1-expressing mutants (Figure 7B–D). Importantly, negative enrichment of ECM and EMT genes was observed in mutant sets compared to wild-type sets when the HTRA expression profile was similar. TCGA STAD patients expressing median levels of HTRA1 showed negative enrichment of cell adhesion molecules (Supplementary Figure 15). We also obtained clinical parameters from the clinical annotation file accompanying the TCGA dataset, including tumor stage and overall survival, for HTRA1-mutated patients (Figure 7A). Importantly, these structural variants with predicted reduced protease activity considerably improved patient survival (Figure 7C), suggesting that the functional state of HTRA is important for driving oncogenic signaling and disease progression regardless of the HTRA expression level. Overall, point mutations in HTRA1 demonstrated strong potential for delaying disease progression.

### Cross-mutational analysis of cancer-associated mutations in *H. pylori* and human HTRA1 reveals comparable shifts in free energy values

**Figure 8.**
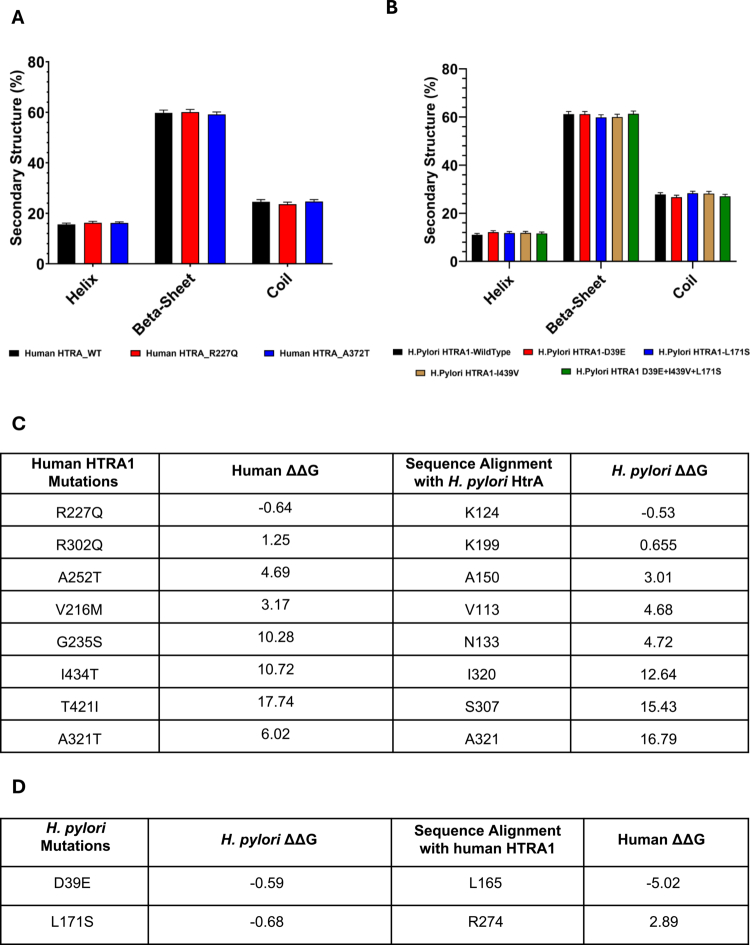
Cross-mutational analysis of human and *H. pylori* HtrA. (A) Bar graphs of averaged secondary structure percentage for Human wild-type, R227Q, and A372T HTRA1 (last 50  ns). (B) Bar graphs of averaged secondary structure percentage for *H. pylori* wild-type, D39E, L171S, I439V, and D39E + L171S + I439V HtrA (last 50 ns). (C, D) Cross-mutational analysis between human STAD-associated mutations in *H. pylori* and *H. pylori* mutations in human HTRA1 reveals comparable free energy values, suggesting a potentially conserved functional role of human and *H. pylori* HtrA in disease progression.

To investigate the possibility of a conserved functional relationship between humans and *H. pylori* HtrA, a secondary structure and sequence-aligned cross-mutational analysis was performed, in which the STAD-associated human variants R227Q and A372T were mapped onto *H. pylori* HtrA and *H. pylori*-derived mutations D39E and L171S were projected onto human HTRA1 (Figure 8). All the analyzed protein structures showed a similar secondary profile, highlighting the potential functional similarity between human and *H. pylori* HtrA proteins despite differences in their protein sequences (Figure 8A-B). Mutational profiling revealed comparable predicted free energy shifts in both human and bacterial HtrA, which indicates similar effects on protein stability and structural dynamics (Figure 8C–D) and suggests that the corresponding residues in human and bacterial HtrA may contribute to shared functional mechanisms relevant to gastric disease progression. The alignment of these energetic alterations implies that both Gastric Adenocarcinoma-linked mutations and bacterial HtrA-related changes may target common structural or functional regions within HTRA1, suggesting that human HTRA1 and *H. pylori* HtrA influences proteolytic regulation, extracellular matrix dynamics, and epithelial barrier function during gastric carcinogenesis through conserved biological mechanisms. Taken together, *H. pylor*i HtrA and human HTRA protease activity is an important feature that modulates oncogenic signaling, with implications for patient survival. Hence, the SNP-based protein variants as well as the expression levels of the host HTRA and bacterial HtrA could be used to stratify patients for survival and treatment outcome.

## Discussion

### STAD analyses revealed typical Wnt signaling (as known from other cancers)

**Figure 9.**
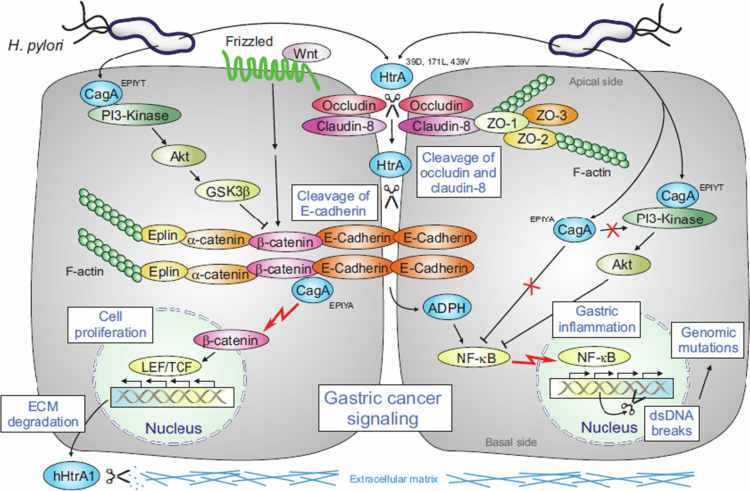
Model showing the role of Wnt signaling to E-cadherin and β-catenin in the development of gastric cancer, which is controlled by SNP variants of *H. pylori* CagA and HtrA, and human HtrA1.

Our gene enrichment analysis of STAD tissue samples revealed transcriptional upregulation of genes involved in several cellular pathways, particularly those associated with cholesterol metabolism, Wnt signaling, complement and coagulation cascades, and microRNA activity in cancer (Figure 3B). The expression of other genes, such as those involved in immune response pathways, such as the differentiation of Th1/Th2/Th17 cells, intestinal immune regulation, and chemokine signaling, was markedly reduced in the analyzed cancer tissue (Figure 3B). Of particular interest is the Wnt signaling pathway, a conserved signaling cascade that is crucial in regulating cell differentiation and proliferation and tissue homeostasis.^54^ The signal protein Wnt plays a central role in the β-catenin signaling pathway.^55^ ^,^ ^56^ When Wnt is absent, β-catenin is phosphorylated by GSK3, which targets β-catenin for ubiquitination and degradation by cellular proteasomes.^40^ ^,^ ^57^ However, binding of Wnt ligands activates disruption of the β-catenin destruction complex, which prevents GSK3 from phosphorylating β-catenin, and thus, allows the migration and accumulation of β-catenin in the nucleus.^53^ Nuclear β-catenin interacts as a co-factor with several transcription factors, including TCF/LEF (T cell factor/lymphoid enhancer factor), to regulate expression of target genes that induce cell differentiation and proliferation (Figure 9) (reviewed in^58^ ^,^ ^59^). Hyperactivation of the canonical Wnt signaling pathway, for example by mutations in components of the destruction complex, is often responsible for the development of tumors, as this complex regulates the amount of free cytosolic β-catenin. When β-catenin is no longer phosphorylated, it can no longer be degraded via the ubiquitin‒proteasome pathway.^54^ ^,^ ^59^ In addition, β-catenin is released from the E-cadherin/β-catenin complex after the cleavage of endothelial cell junctions by the host cell HTRA ^44^ or after the cleavage of epithelial cell junctions by *H. pylori* HtrA^23^, providing an excess of cytosolic β-catenin, which in turn can promote cell proliferation (Figure 9). Thus, carcinomas may be caused by a deregulated Wnt pathway, which controls cell proliferation and metastasis, including GC.^9^ ^,^ ^56^ ^,^ ^60^ ^,^ ^61^ Concurrently, single-cell gene expression analysis showed strong co-expression of the CTNNB1 gene encoding β-catenin, of HTRA1, and of the CCND1 gene encoding cyclin D1 (Figure 5A). Cyclin D1, a protein that regulates the transition of the G1 cell phase into the S phase via activation of cyclin-dependent kinases 4 and 6 (CDK4, CDK6), is often overexpressed in tumor cells.^62^

### Role of human HTRA proteases

Homologs of the HTRA-type of serine proteases are evolutionarily conserved among cellular organisms, and are thus present in bacteria and eukaryotes (Supplementary Figure 3). Human cells express four HTRA homologs, named HTRA1, HTRA2, HTRA3, and HTRA4. They contain a protease domain with the catalytic triad His-Asp-Ser, and a PDZ domain.^44^ ^,^ ^45^ ^,^ ^63^ These serine proteases play crucial roles in the quality control of cellular proteins.

Most of HTRA1 is secreted into the extracellular space, and only part of HTRA1 is located intracellularly, which is mainly attached to microtubules.^64^ ^,^ ^65^ HTRA1 has a wide range of functions, and several targets of HTRA1 have been identified, including growth factors and ECM proteins such as fibronectin, collagen, and decorin.^44^ HTRA1 is involved in the induction of apoptosis and anoikis. Therefore, HTRA1 is considered an important tumor suppressor. Accordingly, the expression of HTRA1 was found to be downregulated in multiple different cancers compared to healthy tissue,^44^ and HTRA1 was found to be only weakly expressed in gastric tumors compared to moderate to strong expression in normal gastric tissue. HTRA1 overexpression was reported to suppress cell proliferation and to prevent cell migration,^66^ collectively suggesting that decreased HTRA1 expression is a general characteristic of cancer. In contrast, our results showed increased HTRA1 transcript levels in samples from STAD patients relative to gastric tissue from healthy controls (Figure 3C), and survival probability curves showed better survival chances of STAD patients with low and medium HTRA1 expression compared to high HTRA1 levels (Figure 3D). The pathway enrichment analyses of high HTRA1 expressing samples revealed differential upregulation of many genes involved in the ECM-receptor interaction pathway (Figure 4C, 4E). Many of those genes encode structural proteins of the ECM, including fibronectin, laminin, collagen, vitronectin, tenascin, and THBS, showing that HTRA1 affects the reorganization of the ECM (Figure 4E). In addition, the analysis showed enrichment of the KRAS signaling pathway, inflammatory responses, and angiogenesis in high HTRA1-expressing patients (Figure 4C). Aberrant RTK–RAS signaling is a well-established feature of GC and can arise through mutations, gene amplification, or upstream receptor activation. KRAS signaling regulates cell proliferation, survival, migration, and metabolism through downstream pathways, including MAPK/ERK and PI3K/AKT kinases.^67^ These down-stream targets can in turn stabilize β-catenin and promote the expression of Wnt target genes involved in cell proliferation and migration. Consistent with this view, PI3K/AKT kinase signaling was enriched among the upregulated genes in patients with high HTRA1 expression (Figure 4C).

Furthermore, increased expression was reported to correlate with upregulated expression levels of alpha smooth muscle actin (*α*-SMA) and of several additional markers for cancer-associated fibroblasts, including *α*-SMA, FAP, FSP-1, CXCL12, PDGFRα and PDGFRβ, indicating that HTRA1 promotes proliferation of normal fibroblasts to cancer-associated fibroblasts.^68^ Indeed, high expression of HTRA1 and HTRA3 was predominantly detected in fibroblasts, in endothelial cells and in cancer cells (Figure 5).In high HTRA1-expressing patients, inflammatory response pathways were positively enriched (Figure 4B), and immune cell infiltration analyses indeed showed significantly higher influx of CD4+ T-cells, CD8+ T-cells, B-cells, macrophages, neutrophils, and mast cells in high HTRA1 expressing patients than in low HTRA1 expressing patients (Supplementary Figure 9). The analysis showed both pro-inflammatory (neutrophils, M1 macrophages), and anti-inflammatory (M2 macrophages) immune cells, indicating a complex inflammatory response (Supplementary Figure 9). However, in both physiological tissue repair and tumor microenvironments, Wnt signaling pathways (particularly the canonical Wnt/β-catenin pathway) promote the polarization of macrophages to the M2 state and enhance the expression of M2 markers while limiting pro-inflammatory M1 responses.^69^ ^,^ ^70^ In addition, persistent or aberrant activation of Wnt signaling in macrophages often contributes to extended M2 dominance, leading to excess ECM deposition.^71^ In agreement with these findings, immune cell infiltration analysis predominantly detected M2 macrophages among the attracted immune cells (Supplementary Figure 9).

HTRA1 protein variants can exacerbate disease progression and thus promote cancer, such as mutations A321T and A372T that resulted in patient survival for less than seven months (Figure 7A, 7C). In contrast, other mutations with positive ΔΔG, that are predicted to structurally weaken the HTRA protein, such as mutation G235S, can be protective and are associated with longer patient survival compared to non-mutant HTRA1 (Figure 7C). Thus, mutations in HTRA1 modulate cancer progression and can either show causative effects or protective effects, crucially influencing patient survival chances.

Similar to HTRA1, HTRA3 is a chaperone and protease that is located extracellularly, but also intracellularly in the cytoplasm and mitochondria. Among the cellular targets of HTRA3 are cytoskeletal proteins such as actin, β-tubulin, vimentin, and TCP1 chaperonin.^72^ Accordingly, the upregulated gene pathways included cell adhesion molecules, focal adhesion, and ECM-receptor interactions (Supplementary Figure 10). In addition, HTRA3 interacts with various transforming growth factor proteins, including TGF-β1, TGF-β2, and growth and differentiation factor (GDF)-5, ^73^ and regulates mitochondria-mediated apoptosis.^74^ HTRA3 is regarded as a tumor suppressor, because its decreased expression has been observed in various cancer tissues,^44^ but in esophageal^75^ and pancreatic adenocarcinoma^76^ HTRA3 expression is increased compared to controls. Here, we show that HTRA3 expression is also increased in STAD tissue compared to healthy controls (Figure 3C), and that relatively high expression levels significantly reduce the survival probability compared to patients with low or moderate HTRA3 expressions (Figure 3E). Similar to HTRA1, HTRA3 expression was high in fibroblast cells (Figure 3G, Figure 5), but not in endothelial cells.

HTRA2 is associated with mitochondria, where it maintains mitochondrial homeostasis, mediating both cell survival and cell death. While HTRA2 was detected in several different cancers, its expression varies depending on the type of cancer.^44^ Our data showed no significant difference in HTRA2 expression in the STAD samples compared to the control group (Figure 3C). HTRA4 is mainly associated with pregnancy and is usually barely to weakly expressed in other cells than in the placenta, including gastric cells.^44^ Although our data indeed showed only a limited number of HTRA4 transcripts in gastric tissue (Figure 3C), the STAD samples exhibited significantly higher HTRA4 expression than the control tissue, suggesting HTRA4 activation.

### Role of *H. pylori* HtrA

Similar to other bacterial HtrAs, *H. pylori* HtrA is composed of a signal peptide, a protease domain with the catalytic triad His-Asp-Ser, and two PDZ domains. *H. pylori* HtrA is mainly localized in the periplasm, where its chaperone function protects periplasmic proteins from environmental stress, including heat stress, and degrades misfolded and otherwise aberrant proteins.^63^ In addition, HtrA plays a crucial role in the interaction of the bacteria with the gastric epithelium. Upon binding to the apical cell surface, which predominantly occurs at the cell junctions of the host epithelium, HtrA is secreted and opens the cell-to-cell junctions through cleavage of the junction proteins occludin, claudin-8, and E-cadherin.^23^ ^,^ ^63^ The proteolytically active HtrA form is a homo-trimer. Trimer stability is substantially influenced by a histidine residue at amino acid position 46 (His46) of the first, Leu171 of the second, and Arg32 of the third HtrA molecule, which are located in close proximity to each other.^23^ ^,^ ^47^ In the 171S HtrA allele version, Ser171 still closely interacts with Arg32, but binding to His46 is greatly reduced, which decreases trimer stability by an estimated 5.6 kJ/mol ^23^ (Figure 1E), and thus, the proteolytic activity of the enzyme. Cleavage of E-cadherin by HtrA not only compromises the structural integrity of the epithelial barrier but also results in the disruption of the E-cadherin/β-catenin complex and β-catenin accumulation in the nucleus, which triggers proliferative responses,^23^ as outlined above. Consequently, the HtrA 171S/L polymorphism was identified to affect disease outcome in *H. pylori* infections, with 171L as the cancer-associated allele (Figure 1D). The 39D/E polymorphism is located in the secretory signal peptide region. This change resulted in only minor perturbation of the protein structure, and thus only marginally affected trimer stability (Figure 1E, F, H). The change is in close proximity to two previously reported proteolytic cleavage sites, H46/D47 and K50/D51, that were shown to be involved in HtrA processing.^77^ Given that both cleavage sites contain the amino acid D, it is tempting to speculate that the 39D/E polymorphism might affect HtrA processing and secretion.

Cross-mutational analyses revealed comparable predicted free energy shifts between human and bacterial HtrA (Figure 8C, D), suggesting similar effects of the corresponding residues in human and bacterial HtrA on protein stability. The alignment of these energetic alterations implies that both STAD-linked mutations and bacterial HtrA-related changes may target common structural or functional regions within HTRA1. This overlap raises the possibility of a conserved biological mechanism through which human HTRA1 and *H. pylori* HtrA influence proteolytic regulation, ECM dynamics, and epithelial barrier function during gastric carcinogenesis. Together, these observations support the concept of a coordinated host–pathogen interaction axis that may contribute to tumor initiation and progression.

### Role of *H. pylori* CagA

Disrupted epithelial cell junctions allow *H. pylori* to transmigrate from the apical to the basolateral side between the cells, where *H. pylori* employs the *cag*PAI-encoded T4SS to inject ADP-heptose and the bacterial oncoprotein CagA into the epithelial cell.^34^ The LPS metabolite ADP-heptose triggers inflammation via activation of the ALKP1→TIFA→NF-κB signal transduction cascade and the release of interleukin 8 (IL-8).^78^ NF-κB-dependent transcription in S-phase cells promotes the formation of RNA/DNA hybrids, and thus replication stress, which in turn induces the formation of DSBs in the host cell chromosomes and introduces genomic mutations.^33^ Upon entering the cell, CagA is phosphorylated at the C-terminal EPIYA motifs by the host Src and Abl kinases.^37^ Phosphorylated CagA interacts with numerous proteins involved in cell signaling pathways, which results in the disruption of regular cellular functions and in changes in cellular morphology, including the induction of cell elongation, the so-called “hummingbird phenotype”. In addition, non-phosphorylated CagA triggers the loss of cell polarity by inhibiting Par1b kinase activity^79^ and upregulates expression of NF-κB and β-catenin, which promote inflammation and cell proliferation, respectively.^80^ In addition, NF-κB-mediated inflammation stimulated DSBs, which results in the accumulation of chromosomal mutations.^38^

The CagA EPIYA motifs were shown to be associated with the pathological outcome of *H. pylori* infection (Figure 1B). Phosphorylated EPIYA-B binds to the PI3-kinase that activates serine/threonine kinase AKT.^36^ This interaction is pronounced in strains that contain EPIYT, but absent in CagA with EPIYA, because the threonine residue can form a side-chain hydrogen bond with Asn417 of the PI3-kinase, which cannot be formed by alanine. Stimulated AKT inhibits GSK3, which results in reduced phosphorylation, and thus, stabilization of β-catenin. In contrast, CagA with the EPIYA variant triggers the release and transfer of β-catenin to the nucleus. Hence, strains with EPIYT CagA exhibit lower NF-κB stimulation and IL-8-mediated inflammation and less β-catenin-stimulated cell proliferation than *H. pylori* carrying the EPIYA allele.^36^ As a result, EPIYA is associated with the development of GC and ulcers (Figure 1B, C), whereas EPIYT represents an attenuated allele.^81^

Thus, in teamwork with CagA, particularly the disease-associated EPIYA-B motif, *H. pylori* serine protease HtrA promotes the development of STAD. The GC-associated variants of *H. pylori* HtrA efficiently open the cell-to-cell junctions by cleavage of junctional proteins occludin, claudin-8, and E-cadherin, thereby releasing β-catenin from the β-catenin/E-cadherin complex (Figure 9). The transport and accumulation of β-catenin into the nucleus induce cell proliferation, a hallmark of STAD development. In addition, disruption of cellular junctions allows bacterial migration from the apical to the basolateral side of the host cells, where *H. pylori* employs the *cag*PAI-encoded T4SS to deliver ADP-heptose and CagA into the host cell ^34^ (Figure 9). After injection, the CagA oncoprotein also targets E-cadherin and further stimulates the β-catenin translocation into the nucleus and subsequent proliferative transcription. While the EPIYA-B motif variant EPIYA promotes CagA-mediated β-catenin transfer and NF-κB activation, the EPIYT variant exhibited a significantly attenuated effect due to the induction of the PI3K/AKT kinase pathway ^36^ ^,^ ^40^ (Figure 9). Together, *H. pylori* HtrA and CagA effectively promote the development of STAD. In addition to *H. pylori*-induced GC, overexpression of human HTRA, particularly HTRA1, also disrupts the balance of the Wnt/β-catenin signaling pathway and damages the ECM by dysregulating the expression of several ECM proteins, including fibronectin,^53^ further promoting disease development and progression. Thus, *H. pylori* HtrA and CagA, as well as human HTRA, stimulate the Wnt/β-catenin signaling pathway and thereby contribute to the development and progression of GC. While strong expression of HTRA1 generally results in a poorer prognosis for patient survival (Figure 3, Figure 7), different HTRA1 variants can either accelerate or slow the progression of the disease (Figure 7). The impact of HTRA mutations on disease progression and severity depends, at least in part, on how the identified polymorphisms affect the structural stability of the proteolytically active HTRA trimers, as discussed above.

Overall, multiple factors have been described to contribute to the development of STAD. These include the genetic predisposition of the patient to specific allele variants in human genes that affect chromosome and microsatellite stability, impair DNA repair and several germline mutations,^9^ the presence and specific variants of *H. pylori* virulence factors,^81^ and environmental factors such as poor diet, alcohol, and smoking.^13^ Here, we explored the role of human HTRA serine proteases in STAD progression and showed elevated expressions of HTRA1 and HTRA3 in stomach cancer samples, in contrast to their decreased expression in cancers of many other organs. High HTRA1 expression levels in STAD samples, particularly in fibroblasts and endothelial cells, coincided with dysregulated ECM interaction and EMT signaling pathways that are known to affect tumor invasion, cell migration, and metastasis. Likewise, HTRA1 and β-catenin co-expression in single cells concurred with increased expression of epithelial cell signaling, Wnt/β-catenin and ECM damage pathway genes in *H. pylori* infected STAD patients. We spanned the arc to *H. pylori* HtrA serine protease and oncoprotein CagA, both of which compromise the Wnt/β-catenin signaling pathway in the stomach epithelium, a key player in STAD progression. We showed that specific HtrA and CagA alleles are associated with gastric cancer development, and so is the overexpression of human HTRA1. All these factors must be considered high-risk factors, especially when combined, contributing to disease development and progression, and highlighting the complex interplay between human and bacterial factors in this important disease. Our data with specific GC-associated SNPs in the *H. pylori* virulence factors CagA and HtrA and ancient human migrations of the bacterium might also explain why the GC incidence in Eastern Europe and East Asia is higher compared to the rest of the world (Figure 1A–D, Figure 2). In summary, SNPs in *H. pylori* and the human serine protease HtrA and *H. pylori* CagA modulate cancer signaling and can be causative or protective factors.

## Materials and methods

### *H. pylori* genomes and gastric disease status

We compiled a genome database of 2,267 *H. pylori* isolates of worldwide origin with known disease status, which included genomes from bacterial genome-wide association studies (GWAS),^82^ ^,^ ^83^ a functional study on the *htrA* SNPs^23^ and additional publicly available genomes with included disease information that were acquired from Genbank (https://www.ncbi.nlm.nih.gov/datasets/genome/?taxon=210). The *htrA* gene sequences, as well as the multi-locus sequence analysis (MLSA) gene sequences of *atpA*, *efp*, *mutY*, *ppa*, *trpC*, *ureI*, and *yphC,* were extracted using blastn. Based on the MLSA sequences, the genomes were assigned to the biogeographic *H. pylori* populations hpAfrica1, hpAfrica2, hpNEAfrica, hpEurope, hpAsia2, and hpEastAsia, using the software Structure, as described.^49^ ^,^ ^50^ Using tblastn, the *H. pylori* genomes were analyzed for the presence of the *cagA* gene and the C-terminal EPIYA-B motif. A total of 1,736 genomes contained the CagA EPIYA-B motif, of which 1,650 contained the EPIYA (1,229 genomes) or EPIYT (421 genomes) allele variants and 86 genomes contained other variants, including ESIYA (39 genomes), ESIYT (19), EPVYT (7), EPVYA (6), ELIYA (5), EPIYV (2), ESIYD (2), ESIYG (1), ESIYS (1), ESVYA (1), EAIYA (1), ELIYT (1), and GSIYD (1). A total of 1,363 genomes were obtained from patients with non-atrophic gastritis (NAG, *n* = 757), gastric cancer (GC, *n* = 282), and peptic ulcer disease (PUD, *n* = 324), which were analyzed further. The remaining genomes were obtained from patients with atrophic gastritis (AG, *n* = 136), metaplasia (*n* = 166), MALT lymphoma (*n* = 19), and from patients with normal gastric mucosa (*n* = 42). The genomes not containing the cagA gene and/or the EPIYA-B motif were from patients with normal mucosa (*n* = 83), and from patients with NAG (*n* = 322), AG (*n* = 14), metaplasia (*n* = 30), MALT lymphoma (*n* = 11), GC (*n* = 19), and PUD (*n* = 52). Pairwise Fisher's exact tests were used to compare the CagA EPIYA/EPIYT (Figure 1C) and HtrA 39D/E, 171L/S, and 439V/I (Figure 1D) allele frequencies across disease groups by running individual Fisher's tests on every pair (https://www.socscistatistics.com/tests/fisher/default2.aspx). *P*-values of *P* < 0.05 were considered as significant. The gene and protein phylogenies were constructed as neighbor-joining trees in MEGA version X.^84^

### Phylogeny analysis

The *htrA* genes and proteins of 11 bacteria associated with different forms of cancer: *Salmonella enterica* serovar Typhi*, Chlamydia pneumoniae, Escherichia coli, Streptococcus anginosus, Capnocytophaga gingivalis, Prevotella melaninogenica, Streptococcus mitis, Helicobacter pylori, Chlamydia trachomatis, Porphyromonas gingivalis*, and *Campylobacter jejuni*, as well as man, mouse, rat, and zebrafish, were collected from UniProt and NCBI. The sequences were aligned using MEGA12. The neighbor-joining tree of the HTRA proteins was constructed using the Poisson-corrected amino acid distances model with pairwise deletion gaps.

### Protein modeling

The crystal structure and sequence of the HtrA crystallized protein were downloaded from the Protein Data Bank (PDB) [PDB ID: 7XS0 and 3TJN]. The missing residues in the structure were modeled using UniProt sequences, and a 3D model of the protein was constructed using the SWISS-MODEL (https://swissmodel.expasy.org) and Alfa-Fold (https://alphafoldserver.com/welcome) via homology modeling. Templates were ranked according to sequence coverage and similarity from the MODEL Template Library (MTL). The template with 100% sequence identity was selected. The local and global accuracy of the structure were predicted using the QMEAN and the GMQE scores, respectively.

### Free energy calculation

To understand the difference between the wild-type and the mutated HtrA protein in terms of energy, we calculated the free energy change using the FoldX-YASARA software (24.4.29). Three mutations in the *H. pylori* HtrA protein were identified, namely, D39E, L171S, and I439V. These mutations were then generated in the modeled crystal structure individually and in combination using the YASARA software. The Vanderwal clash was changed to 1 to account for optimal packing of the trimer structure and minimize the steric strain. The changes in free energy were then calculated. The same methodology was used to calculate the free energy change of the SNPs in the human HTRA1 gene. These SNPs were collected from the CBioPortal (https://www.cbioportal.org/) database.^85^ Mutations were induced in the structure using the YASARA software. Energy calculations were performed for the wild-type and the mutated structures.

### Molecular dynamics simulations

To investigate the structural and dynamic effects of point mutations in HTRA1 (Human and *H. pylori*), all-atom molecular dynamics (MD) simulations were carried out using GROMACS. The mutant and repaired structures generated through FoldX were solvated in a TIP3P water box with a size of 1.5 nm in all spatial directions, and system neutrality was achieved by adding appropriate Cl^-^ counterions. The simulations were performed using the AMBER99SB-ILDN force field in combination with the TIP3P water model. Each system underwent energy minimization for 50,000 steps using the steepest descent algorithm, followed by sequential equilibration phases: 100 ps under NVT conditions with a V-rescale thermostat at 300 K and 100 ps under NPT conditions at 1 atm using the Parrinello–Rahman barostat. During equilibration, heavy atoms were position-restrained, and hydrogen bonds were constrained using the LINCS algorithm, while long-range electrostatics were treated via the particle mesh Ewald method. Subsequently, a 500 ns production run was conducted under NPT conditions without positional restraints, using a 2 fs integration time step and saving trajectories every 2 ps. Structural stability and conformational changes were assessed through RMSD, RMSF, secondary structure analysis (via STRIDE in VMD), residue interaction mapping, distance measurements, and φ–ψ angle evaluations. The secondary structure elements were categorized into β-sheet components (E, T, B), helices (H, G, I), and coils (C). While full trajectories were used for RMSD and secondary structure profiling, statistical analyses were performed on the final 100 ns (dimer) and 50 ns (protomer) segments. Visualization and plotting were completed using VMD and Grace software.

### Data set extraction and processing

Transcriptomic datasets of 414 stomach adenocarcinoma (STAD) patients and 172 matched benign controls were downloaded from the Broad Institute GDAC (gdac.broadinstitute.org) and UCSC Xena (xena.ucsc.edu).^1^ ^,^ ^86^ Clinical information for STAD patients was obtained from cBioPortal (cbioportal.org) and the Broad GDAC.^85^ Patient identifiers were matched to clinical metadata using Microsoft Excel. Independent validation datasets were identified from the Gene Expression Omnibus (GEO): GSE202165^87^, GSE233973^88^, and GSE27411^89^. Low-expression genes and samples were filtered out using default settings of express analyst^90^ (Threshold of 5, variance filter of 15, Sum Low Abundance Method). The gene IDs were converted to standard formats such as common gene names or Entrez gene identifiers. Normalized expression values were log-transformed and clustered into different groups using Principal Component Analysis to assess the quality of the datasets. The classification of patients according to *H. pylori* infection status was based on the clinical annotation file accompanying the TCGA dataset, which contains information related to *H. pylori* infection status for individual patients. Given that the original TCGA study described *H. pylori* only as sporadic evidence detected across the cohort, the *H. pylori*-positive dataset represents these clinically annotated cases and is not based on an independent microbial detection analysis.

### Differential gene expression studies in patients

Differential gene expressions for the dataset were processed using tools such as iDEP (bioinformatics.sdstate.edu/idep96),^91^ Express Analyst, and the GEO2R tool. The differential gene expression packages used include Linear Models for Microarray Data (LIMMA) or DESeq2 (Negative Binomial Distribution Method), depending on the nature of the dataset. For the microarray datasets, differential expression was carried out using the GEO2R plugin directly provided by the NCBI Gene Expression Omnibus. Both Adj. *P*. Value/FDR of 0.05 and a 2-fold change filter of ±1 was applied on the dataset to identify differentially expressed genes. Individual gene expression plots were generated using GraphPad Prism (10.4.2). The sample size used for the different datasets is directly provided in the figures. Unpaired non-parametric Mann-Whitney U test was used to compute the statistical significance.

### Downstream analyses (pathway, correlation and survival, kinase enrichment, immune filtration) in patients

To compute the biological significance, the differentially expressed genes were binned into biological pathways. The datasets were grouped into tumor and benign, high and low HTRA-expressing patients (based on median values), and finally, mutant and non-mutants. Samples were additionally stratified by *H. pylori* infection status according to clinical annotations in the TCGA dataset. Gene Set Enrichment Analysis (GSEA) and pathway over-representation analysis were performed using GDAC workflows: iDEP (bioinformatics.sdstate.edu/idep96), Enrichr, KEGG, and Hallmark^92^ databases. Only pathways with an FDR threshold of < 0.05 were considered significant. The significant genes were further mapped to the KEGG pathway network.^93^ ^,^ ^94^ Survival analyses were performed using UALCAN (ualcan.path.uab.edu).^95^ Common pathways across datasets were identified using Venny (bioinfogp.cnb.csic.es/tools/venny). The sample sizes used for the different datasets are directly provided in the corresponding figures. Kinase enrichment analysis was carried out using https://maayanlab.cloud/X2K/ across different datasets. Briefly, this method identifies upstream regulatory networks by integrating enrichment of significant genes based on chromatin immunoprecipitation (ChIP)-seq/ChIP-chip data, position weight matrices (PWMs), protein–protein interaction networks, and kinase–substrate phosphorylation relationships.^96^ Associations between copy number variations and HTRA1 expression in STAD were obtained from https://www.cbioportal.org/. TIMER ^97^ was used to estimate immune cell infiltration in the tumor microenvironment. To validate the immune infiltration results, five independent algorithms, TIMER, EPIC, CIBERSORT, CIBERSORT-ABS, and xCell, were applied.^97^

### Proteomic data analysis

The CPTAC STAD proteomic dataset was analyzed using https://cppa.site/cppa/. The significant proteins were obtained with a fold change of 1.2 and an Adj. *P*. value of 0.01. These genes were binned into pathways using the Biological Pathways and Hallmark databases.^98^

### Single-cell analysis

Single-cell datasets GSE134520^99^, GSE210347^100^, and GSE167297^101^ were analyzed using Single Cell Cancer Explorer (bianlab.cn/scCancerExplorer/home). The portal provides a simple, direct, and GUI-based approach for generating UMAP, gene expression, and enrichment ratio from Single Seq datasets pertaining to different cancers.^102^ Single-cell datasets were selected based on the literature. Genes of interest were identified, and the corresponding expression plots were generated.

### Statistics

Statistics were performed by either using Student's t-test or Fisher's exact test. Statistical significance was considered significant with *p* values *p* ≤ 0.05 (*), *p* ≤ 0.01 (**), and *p* ≤ 0.001 (***) or non-significant (n.s.) with *p* values *p* > 0.05.

## Supplementary Material

Supplementary Material Supplementary Material

Suppl Figures.pdf

## Data Availability

All sources of human data are listed in the Materials and Methods. All bacterial genome sequences used are publicly available (https://www.ncbi.nlm.nih.gov/datasets/genome/?taxon=210). The authors confirm that all data supporting the findings of this study are shown in the figures and are described in the text.
