# Supplementary material for: Host and Helicobacter pylori HtrA protease variants converge on Wnt/β-catenin signaling to drive stomach adenocarcinoma
Source: Gut Microbes. 2026 Jul 23;18(1):2704244. doi: 10.1080/19490976.2026.2704244 (PMC13418485; doi:10.1080/19490976.2026.2704244)
Supplement: Supplementary Material [file KGMI_A_2704244_SM7146.docx]

**Supplementary Figures**

**Supplementary Figure 1**. Root Mean Square Deviation (RMSD) profile of *H. pylori* wild-type and mutant HtrA protein during 300 ns molecular dynamics simulation.

**Supplementary Figure 2**. Molecular dynamics simulation of the Root Mean Square Fluctuation (RMSF) profile of *H. pylori* wild-type and mutant HtrA protein (last 20 ns).

**Supplementary Figure 3.** Neighbour-Joining phylogenetic analysis shows a strong similarity between human and *H. pylori* HtrA proteins (Poisson-corrected amino acid distances with pairwise deletion of gaps. Node support was assessed by 100 bootstrap replicates).

**Supplementary Figure 4.** A) Box plots representing tumor grades with human HTRA transcript levels in primary tissues of Stomach Adenocarcinoma (STAD) tumors compared to benign tissue in the TCGA dataset [ (**** P <0.0001, Mann Whitney U test)]. B) Survival curve of human HtrA2 and HtrA4 in STAD patients [Wilcoxon test].

**Supplementary Figure 5.** A-B) UMAP and Bar graph representing different cell types in the STAD single cell dataset (GSE210347). C-D) Violin plot representing human HtrA2 and HtrA4 expression levels in different cell types obtained from STAD single cell dataset (GSE210347). The figures were generated using Single Cell Cancer Explorer (bianlab.cn/scCancerExplorer/home). E-F) UMAP and Bar graph representing different cell types in the STAD single cell dataset (GSE134520).

**Supplementary Figure 6.** A-B) UMAP and Bar graph representing different cell types in the Stomach Adenocarcinoma single cell dataset (GSE167297). C-D) Violin plot representing HTRA1, HTRA2, HTRA3 and HTRA4 expression levels in different cell types obtained from Stomach Adenocarcinoma single cell dataset (GSE167297). The figures were generated using Single Cell Cancer Explorer (bianlab.cn/scCancerExplorer/home).

**Supplementary Figure 7.** Proteomic Analysis of STAD datasets show significant enrichment of GSK3B kinase that is critical for Wnt-β-catenin signaling. A-B) Pie chart and volcano plot showing differentially expressed genes obtained from proteomic STAD dataset analysis (https://cppa.site/cppa/). C) Bar graph showing results obtained from Kinase enrichment analysis of significant genes obtained from proteomic analysis (https://maayanlab.cloud/X2K/).

**Supplementary Figure 8.** GSK3B was found to be commonly enriched across transcriptomic analyses of high HTRA1-expressing STAD patients, *H. pylori*-infected STAD patients, and proteomic analyses of STAD datasets. A) Bar graph showing results obtained from Kinase enrichment analysis of significant genes obtained from comparing High and low HTRA1 expressing STAD patients (https://maayanlab.cloud/X2K/). B) Bar graph showing results obtained from Kinase enrichment analysis of significant genes obtained from comparing *H. pylori*-infected and non-infected STAD patients (https://maayanlab.cloud/X2K/). C) Venn diagram and table representing top ten common kinases enriched between high HTRA1-expressing STAD patients, *H. pylori*-infected STAD patients, and proteomic analyses of STAD datasets (https://bioinfogp.cnb.csic.es/tools/venny/). D, E) Venn diagram (D) and table (E) showing the kinases commonly enriched between high HTRA1-expressing STAD patients, *H. pylori*-infected STAD patients, and proteomic analyses of STAD datasets (https://bioinfogp.cnb.csic.es/tools/venny/).

**Supplementary Figure 9.** Tumor infiltration analysis of STAD datasets showed increased influx of CD4 T-cells, Macrophages, Neutrophils, endothelial cells and fibroblasts in High HTRA1 STAD patients. Summary of the findings of tumor infiltration studies of High (n=100) and Low HTRA1 (n=100) STAD patients using TIMER, EPIC, CIBERSORT, CIBERSORT ABS and XCELL algorithms.

**Supplementary Figure 10.** A) Bar graphs representing significant genes obtained from differential gene expression between high and low human HTRA3-expressing STAD tumors from TCGA patients (High HTRA3 patients (226), Low HTRA3 patients (224), Adjusted FDR<0.05, Log FC ±1). B) Significantly enriched pathways (FDR ≤ 0.05) identified by Gene Set Enrichment Analysis (GSEA) of high and low HtrA3 expression groups in STAD tumors based on data from the Hallmark Database. C-D) Bar graphs summarizing significantly enriched pathways (FDR ≤ 0.05) identified by Pathway Enrichment Analysis comparing high and low HTRA3 expression groups in STAD tumors, using the KEGG Database. E) Visualization of the Wnt signaling pathway (KEGG) overlaid with significantly expressed genes derived from differential analysis between high and low HTRA3 expression groups in TCGA STAD samples. Green represents down-regulated genes (-1) and red represents up-regulated genes (1).

**Supplementary Figure 11.** A) Bar graphs representing significant genes obtained from differential gene expression between high and low human HTRA2-expressing STAD tumors from TCGA patients (High HTRA2 patients (226), Low HTRA2 patients (224), Adjusted FDR<0.05, Log FC ±1). B) Significantly enriched pathways (FDR ≤ 0.05) identified by Gene Set Enrichment Analysis (GSEA) of high and low HTRA2 expression groups in STAD tumors, using the Hallmark Database. C-D) Bar graphs summarizing significantly enriched pathways (FDR ≤ 0.05) identified by Pathway Enrichment Analysis comparing high and low HTRA2 expression groups in STAD tumors, using the KEGG Database. E) Visualization of the ECM-receptor interaction signaling pathway (KEGG) overlaid with significantly expressed genes derived from differential analysis between high and low HTRA2 expression groups in TCGA STAD samples. Green represents down-regulated genes (-1) and red represents up-regulated genes (1).

**Supplementary Figure 12.** A) Bar graphs representing significant genes obtained from differential human gene expression between high and low HTRA4 expressing Stomach Adenocarcinoma (STAD) tumors from TCGA patients (High HTRA4 patients (226), Low HTRA4 patients (224), Adjusted FDR<0.05, Log FC ±1). B) Bar graphs summarizing significantly enriched pathways (FDR ≤ 0.05) identified by Gene Set Enrichment Analysis (GSEA) comparing high and low HTRA4 expression groups in STAD tumors, using the Hallmark Database. C-D) Bar graphs summarizing significantly enriched pathways (FDR ≤ 0.05) identified by Pathway Enrichment Analysis comparing high and low HTRA4 expression groups in STAD tumors, using the KEGG Database. E) Visualization of the Wnt signaling pathway (KEGG) overlaid with significantly expressed genes derived from differential analysis between high and low HTRA4 expression groups in TCGA STAD samples. Green represents down-regulated genes (-1) and red represents up-regulated genes (1).

**Supplementary Figure 13.** Single Cell analysis showing upregulation of angiogenesis and tubulation markers in tumor cells, endothelial cells, monocytes and fibroblasts. The dot plots represent the expression levels of tubulation and angiogenesis markers.

**Supplementary Figure 14.** RMSD (A) and secondary structure analysis (B) of human HTRA1 wild-type and mutants.

**Supplementary Figure 15.** A-B) Bar graphs summarizing significantly enriched pathways (FDR ≤ 0.05) identified by Gene Set Enrichment Analysis (GSEA) comparing mutants and median HTRA1 expression patients in STAD tumors, using the KEGG Database.
