# Supplementary material for: Host and Helicobacter pylori HtrA protease variants converge on Wnt/β-catenin signaling to drive stomach adenocarcinoma
Source: Gut Microbes. 2026 Jul 23;18(1):2704244. doi: 10.1080/19490976.2026.2704244 (PMC13418485; doi:10.1080/19490976.2026.2704244)
Supplement: Suppl Figures.pdf [file KGMI_A_2704244_SM4030.pdf]

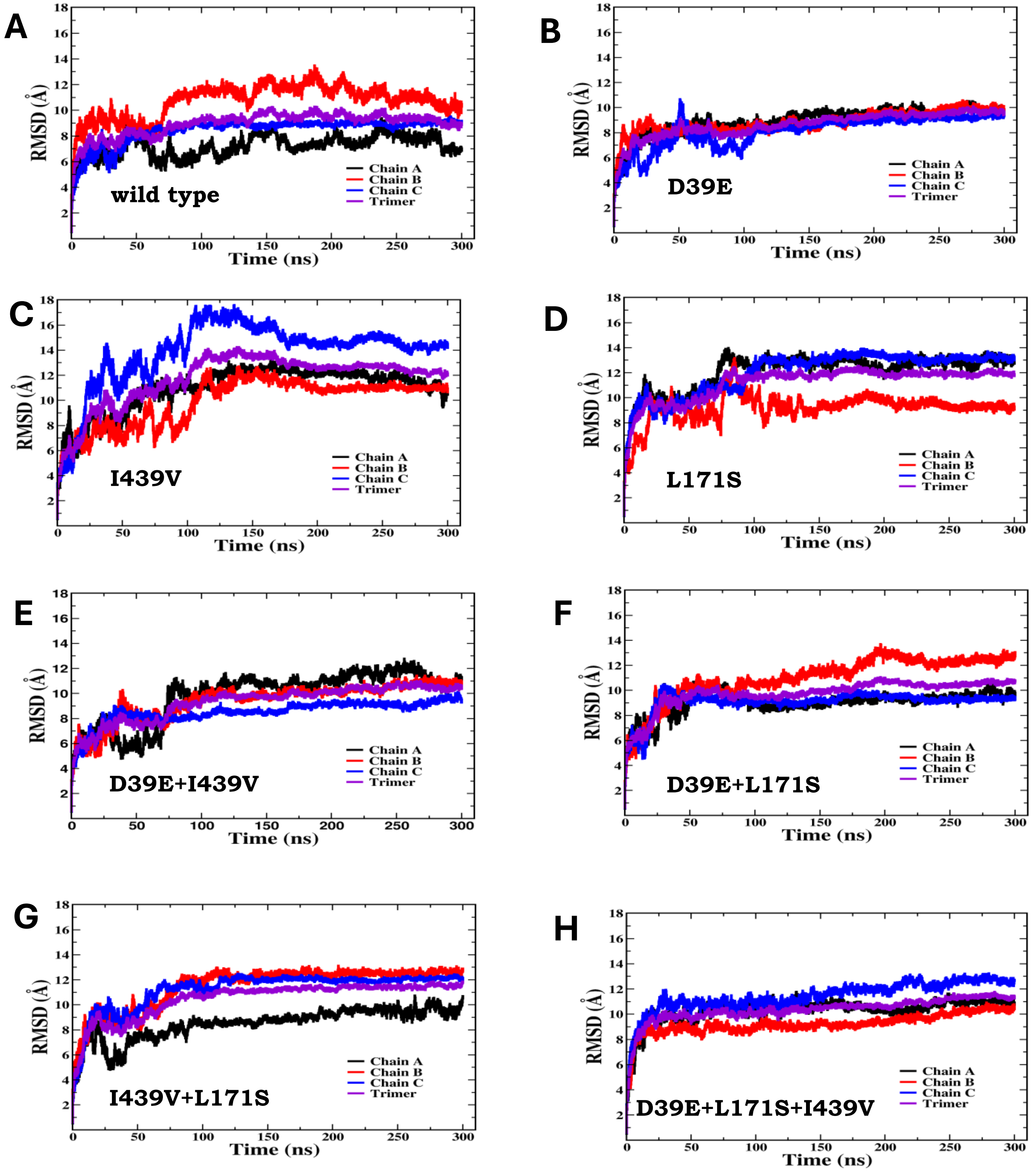

Supplementary Figure 1

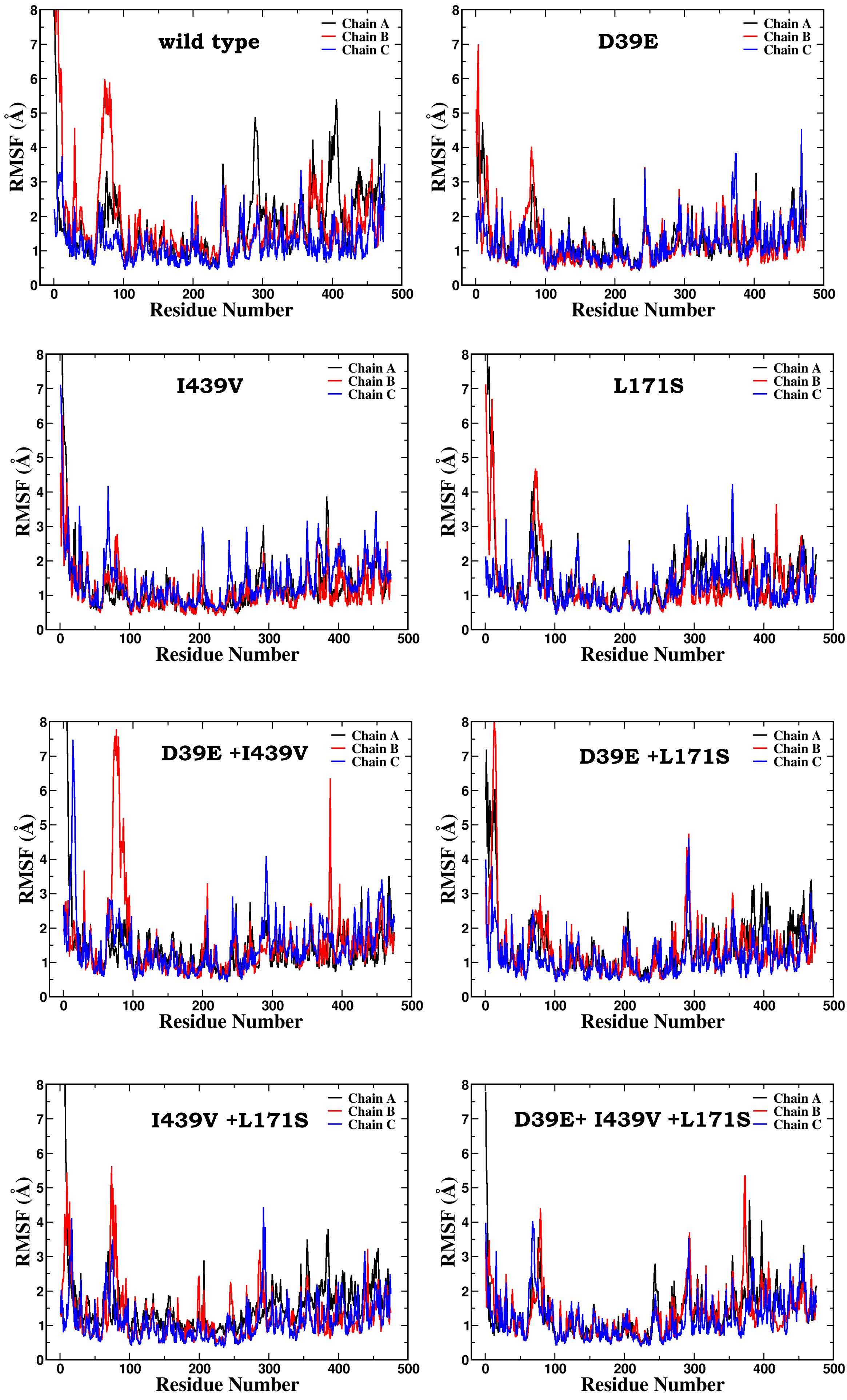

Supplementary Figure 2

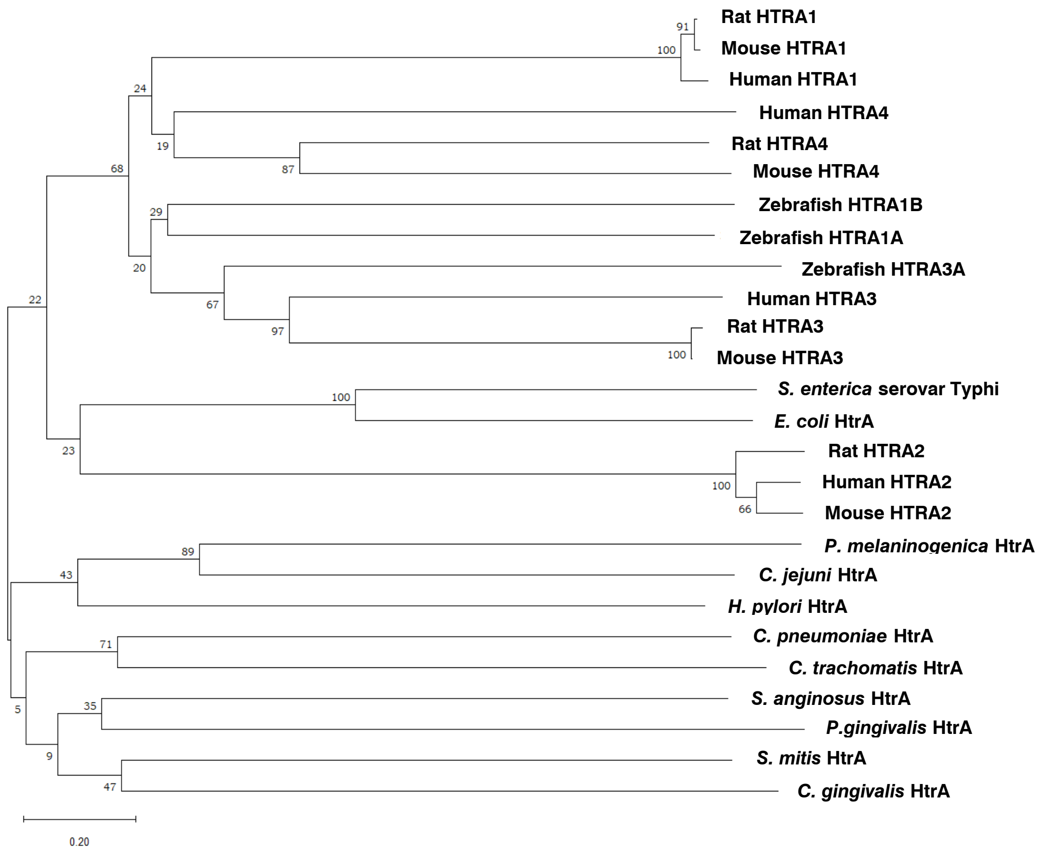

Supplementary Figure 3

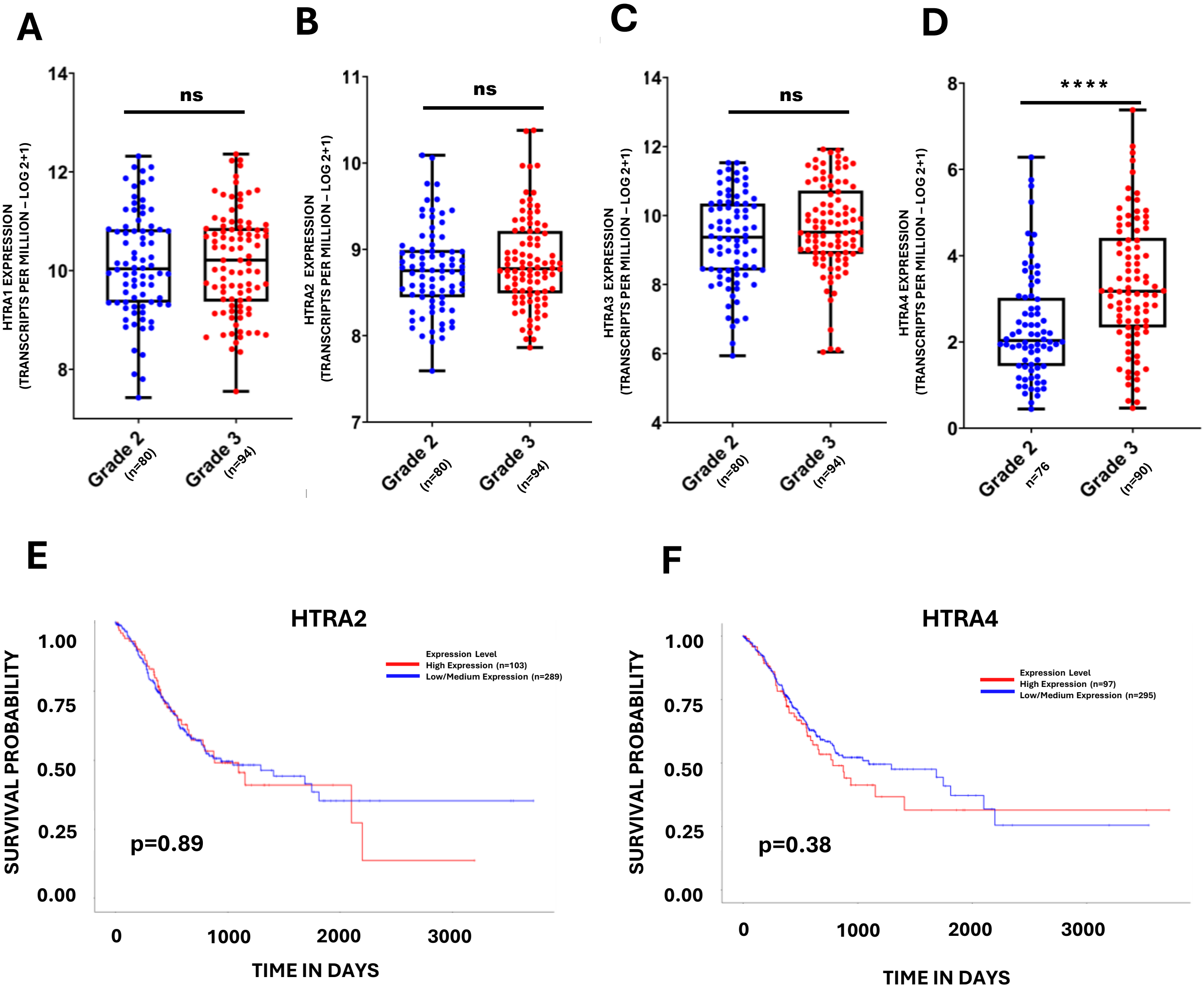

Supplementary Figure 4

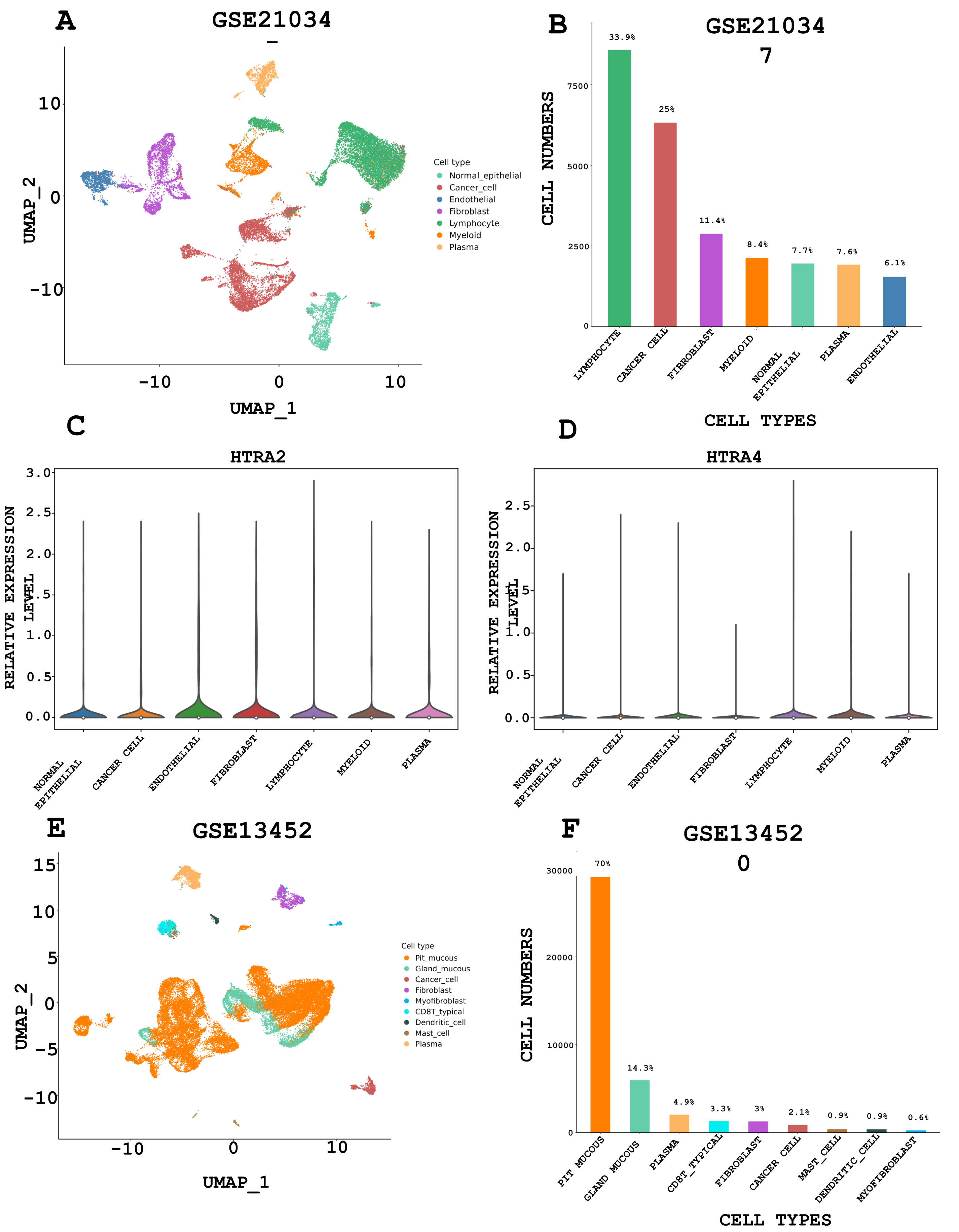

Supplementary Figure 5

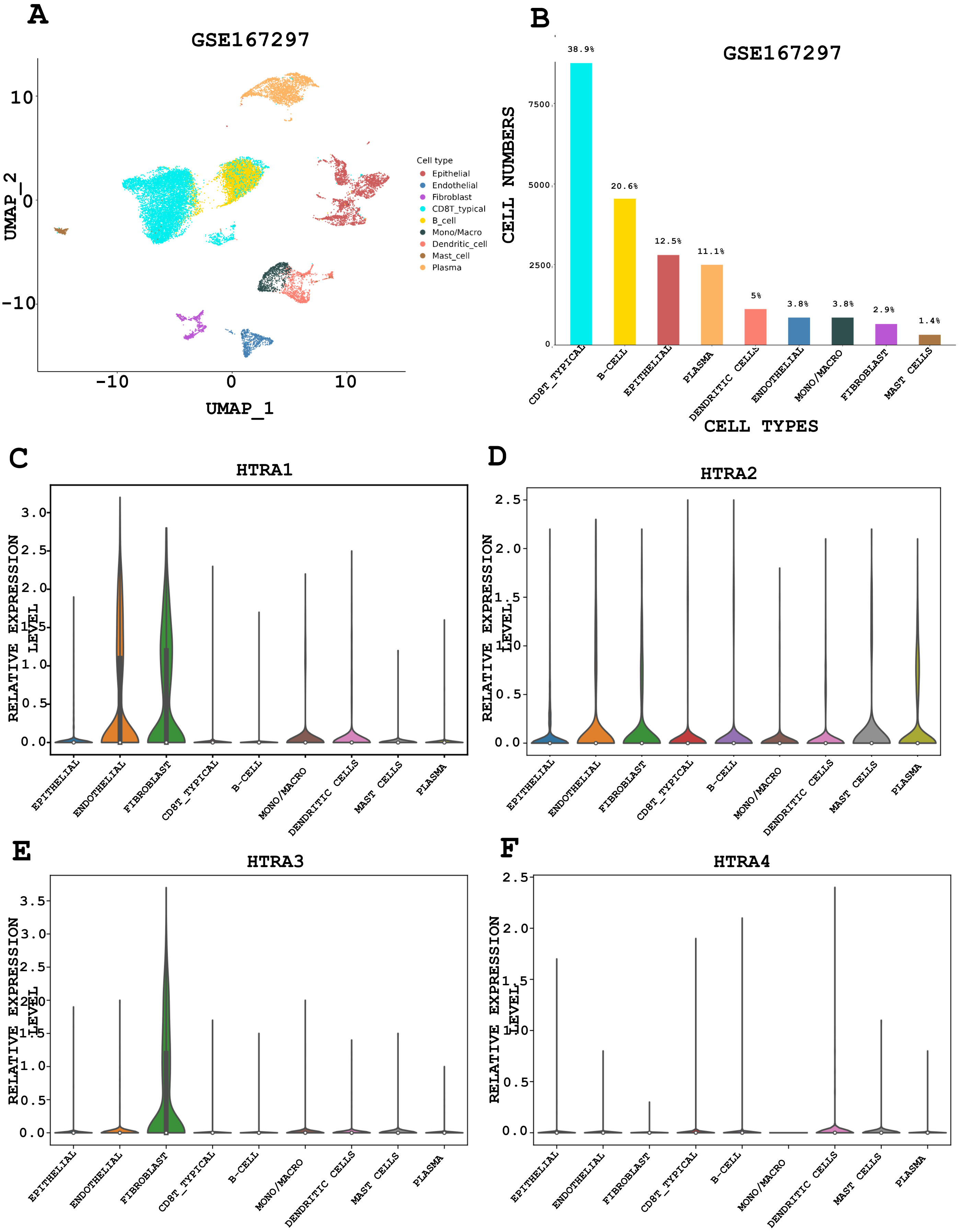

Supplementary Figure 6

STAD PROTEOMICS

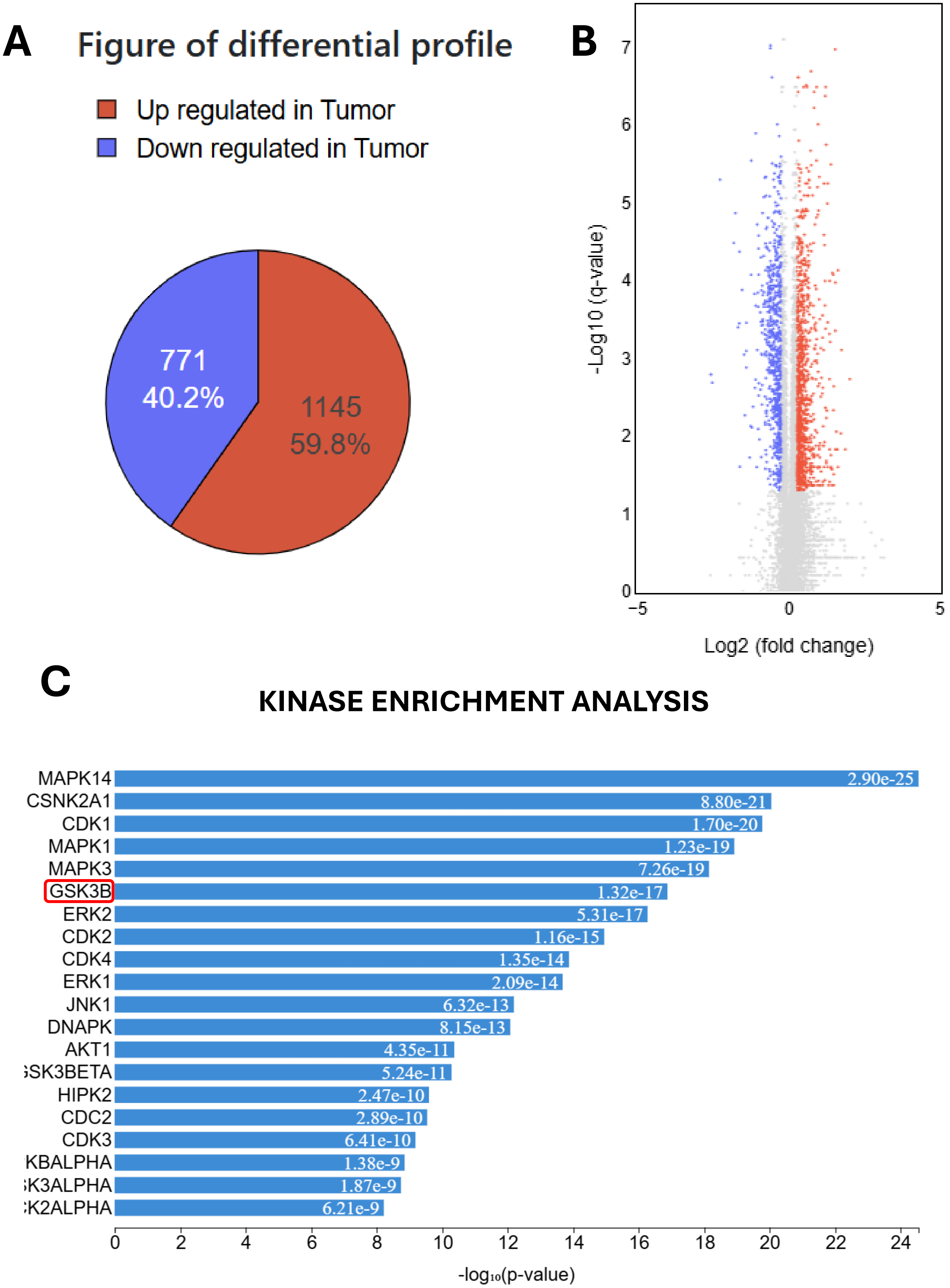

Supplementary Figure 7

A HTRA1 HIGH VS LOW STAD PATIENTS (KINASE ENRICHMENT ANALYSIS)

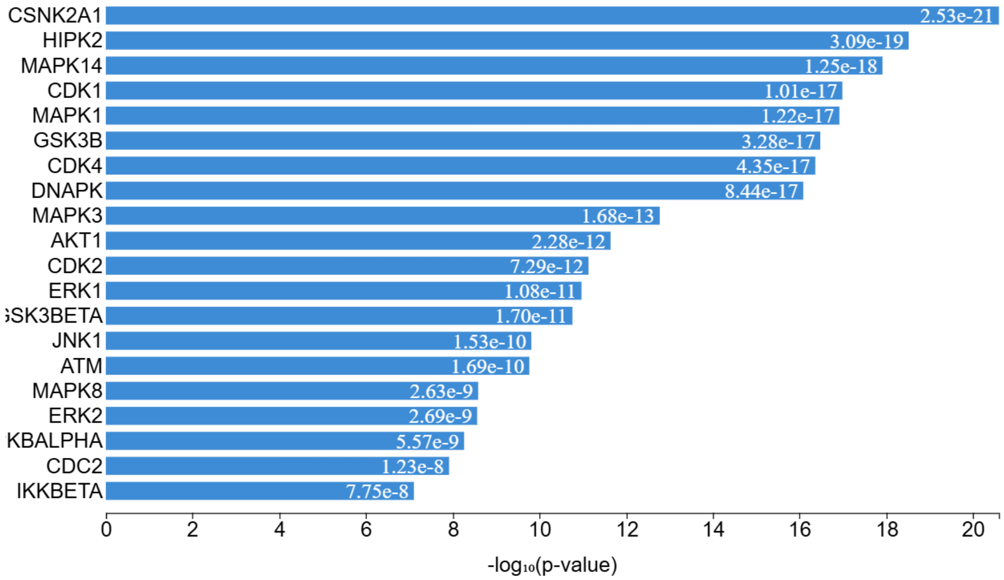

B H. PYLORI INFECTED VS NON-INFECTED (CTRL) STAD PATIENTS (KINASE ENRICHMENT ANALYSIS)

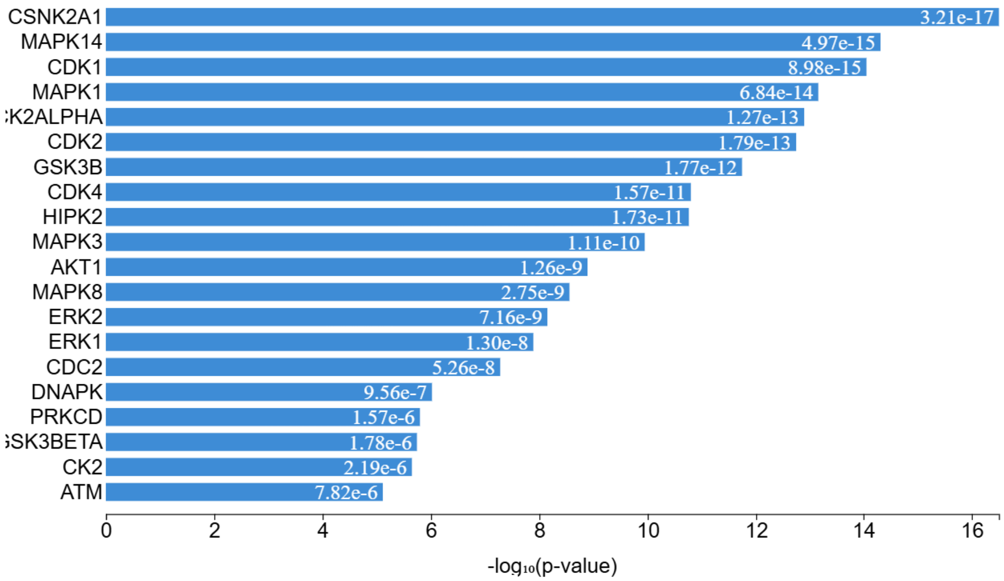

C

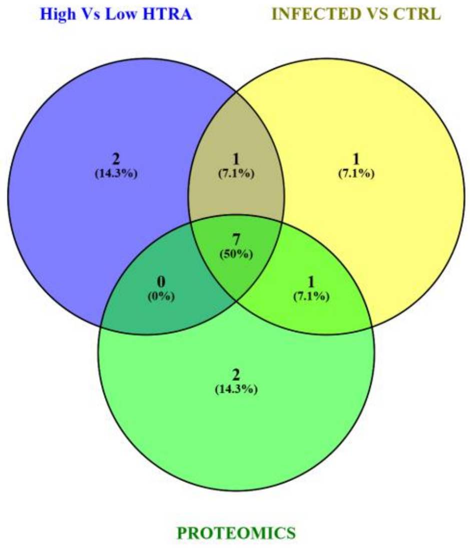

COMMON KINASES (TOP 10)
CSNK2A1
MAPK14
CDK1
MAPK1
**GSK3B**
CDK4
MAPK3

Supplementary Figure 8

D

COMMON KINASES

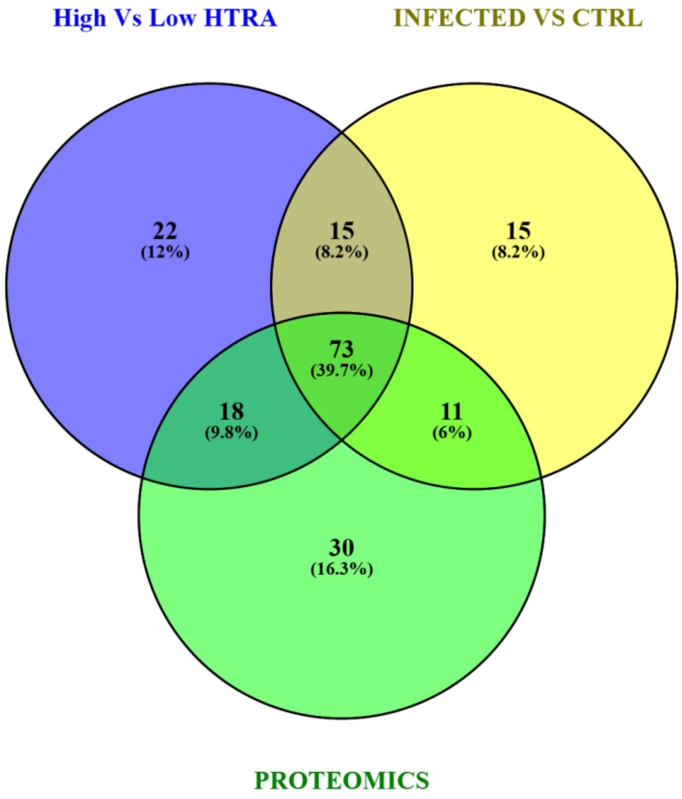

E

CSNK2A1	CHEK2	JNK2	PKA-A
HIPK2	IKKALPHA	PKCTHETA	MAP2K4
MAPK14	CDK5	PIM1	AURORAA
CDK1	TGFBR2	CDK9	MAPK10
MAPK1	CK2ALPHA	PRKCB	PKD1
GSK3B	PRKCZ	AURKA	CSNK2A2
CDK4	RPS6KA3	DNA-PK	ALK
DNAPK	ABL1	CHEK1	MTOR
MAPK3	GSK3ALPHA	PRKACA	CDK7
AKT1	PRKCD	CK2-A	FOXO3
CDK2	TBK1	GSK-3-B	PIKFYVE
ERK1	SRC	PRKAA1	LYN
GSK3BETA	PKBGAMMA	IKBKE	JAK2
JNK1	PRKDC	RPS6KA1	
ATM	PKBBETA	CHUK	
MAPK8	RAF1	CK1ALPHA	
ERK2	CSNK1A1	MAPK9	
PKBALPHA	CSNK1E	RET	
CDC2	PRKCA	PLK1	
IKKBETA	CDK3	IKK-A	

Supplementary Figure 8

TUMOR INFILTRATION IN HIGH (n=100) AND LOW HTRA1 (n=100) STAD PATIENTS

CELLS	TIMER	EPIC	CIBERSORT	CIBERSORT-ABS	XCELL
**CD4**	SIGNIFICANT (P=8.42E-03)	SIGNIFICANT (P=5.55E-02)	SIGNIFICANT CD4 MEMORY ACTIVATED (P=2.78E-02)	SIGNIFICANT CD4 MEMORY RESTING (P=1.12E-05)	SIGNIFICANT CD4 MEMORY (P=5.23E-05)
**CD8**	SIGNIFICANT (P=1.75E-03)	N.S.	N.S.	SIGNIFICANT (P=4.93E-02)	CD8 NAÏVE (TENDING TOWARDS SIGNIFICANCE)
**B-CELLS**	N.S.	SIGNIFICANT (P=3.45E-02)	NIL	SIGNIFICANT (P=4.52E-02)	N.S.
**MONOCYTES**	N.S.	N.S.	SIGNIFICANT (P=2.96E-02)	SIGNIFICANT (P=9.74E-04)	SIGNIFICANT (P=7.94E-05)
**MACROPHAGES**	SIGNIFICANT (P=1.98E-25)	SIGNIFICANT (P=2.21E-05)	SIGNIFICANT MACROPHAGES M2 (P=3.09E-13)	SIGNIFICANT MACROPHAGES MO (P=6.87E-02) MACROPHAGES M2 (P=1.96E-14)	SIGNIFICANT MACROPHAGES M1 (P=1.27E-02) MACROPHAGES M2 (P=8.89E-05)
**MAST**	N.S.	N.S.	SIGNIFICANT (P=3.34E-06)	SIGNIFICANT ACTIVATED MAST (P=1.18E-06)	SIGNIFICANT (P=3.59E-04)
**MYELOID DENDRITIC CELLS**	SIGNIFICANT (P=4.45E-07)	N.S.	N.S.	N.S.	SIGNIFICANT (P=1.75E-02)
**ENDOTHELIAL CELLS**	N.S.	SIGNIFICANT (P=1.56E-26)	N.S.	N.S.	SIGNIFICANT (P=3.62E-23)
**FBROBLASTS**	N.S:	SIGNIFICANT (P=3.60E-33)	N.S.	N.S.	SIGNIFICANT (P=1.32E-31)
**T CELL FOLICULAR HELPER**	N.S:	N.S.	SIGNIFICANT (P=7.46E-07)	N.S:	N.S.
**T CELL REG**	N.S.	N.S.	SIGNIFICANT (P=1.49E-04)	N.S.	N.S.
**NEUTROPHIL**	SIGNIFICANT (P=3.31E-09)	N.S.	N.S.	N.S.	SIGNIFICANT (P=1.57E-02)
**EOSINOPHIL**	N.S.	N.S.	N.S:	N.S:	SIGNIFICANT (P=1.85E-02)
**NK CELLS**	N.S.	N.S:	SIGNIFICANT NK RESTING (P=2.43E-03)	N.S.	N.S:

Supplementary Figure 9

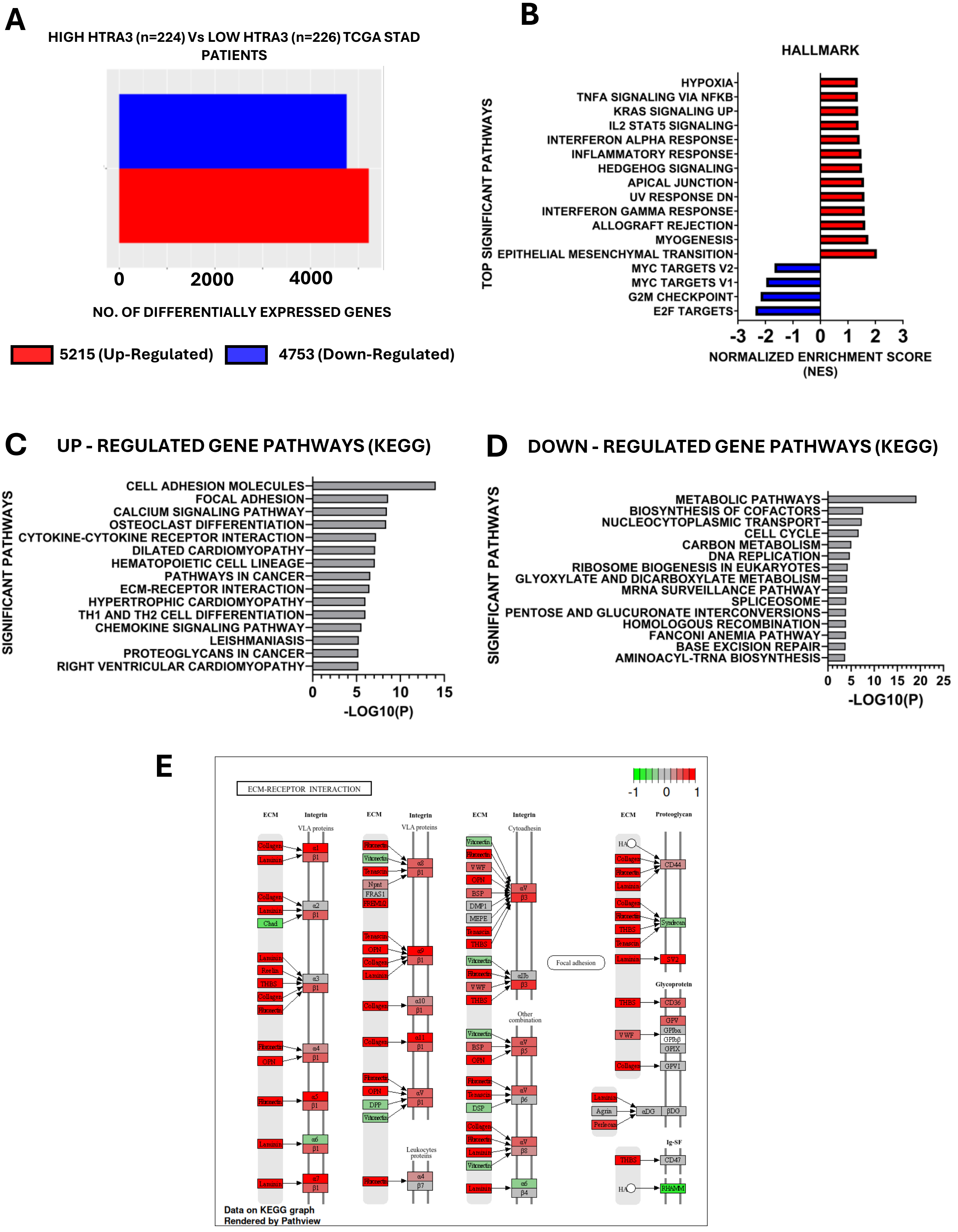

Supplementary Figure 10

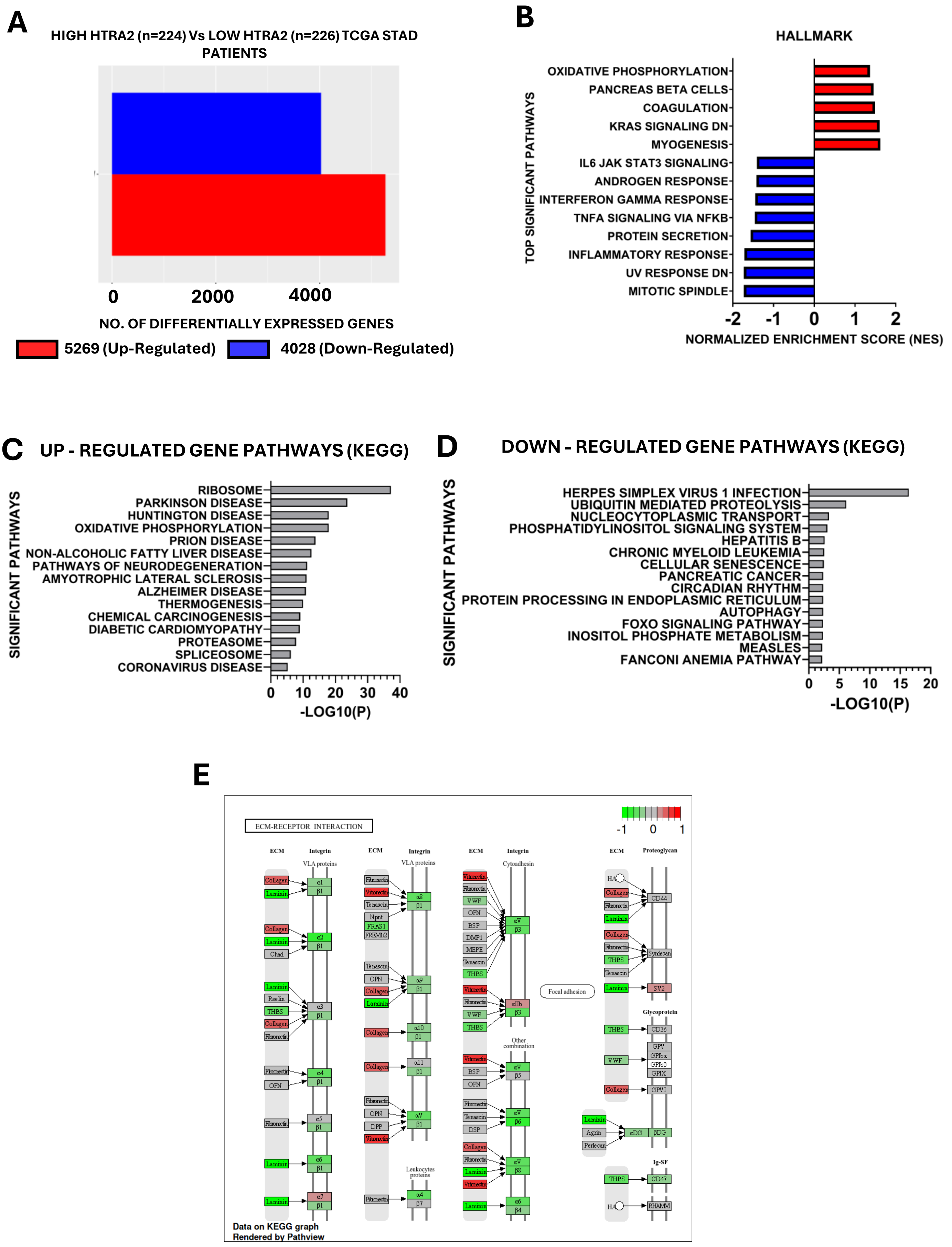

Supplementary Figure 11

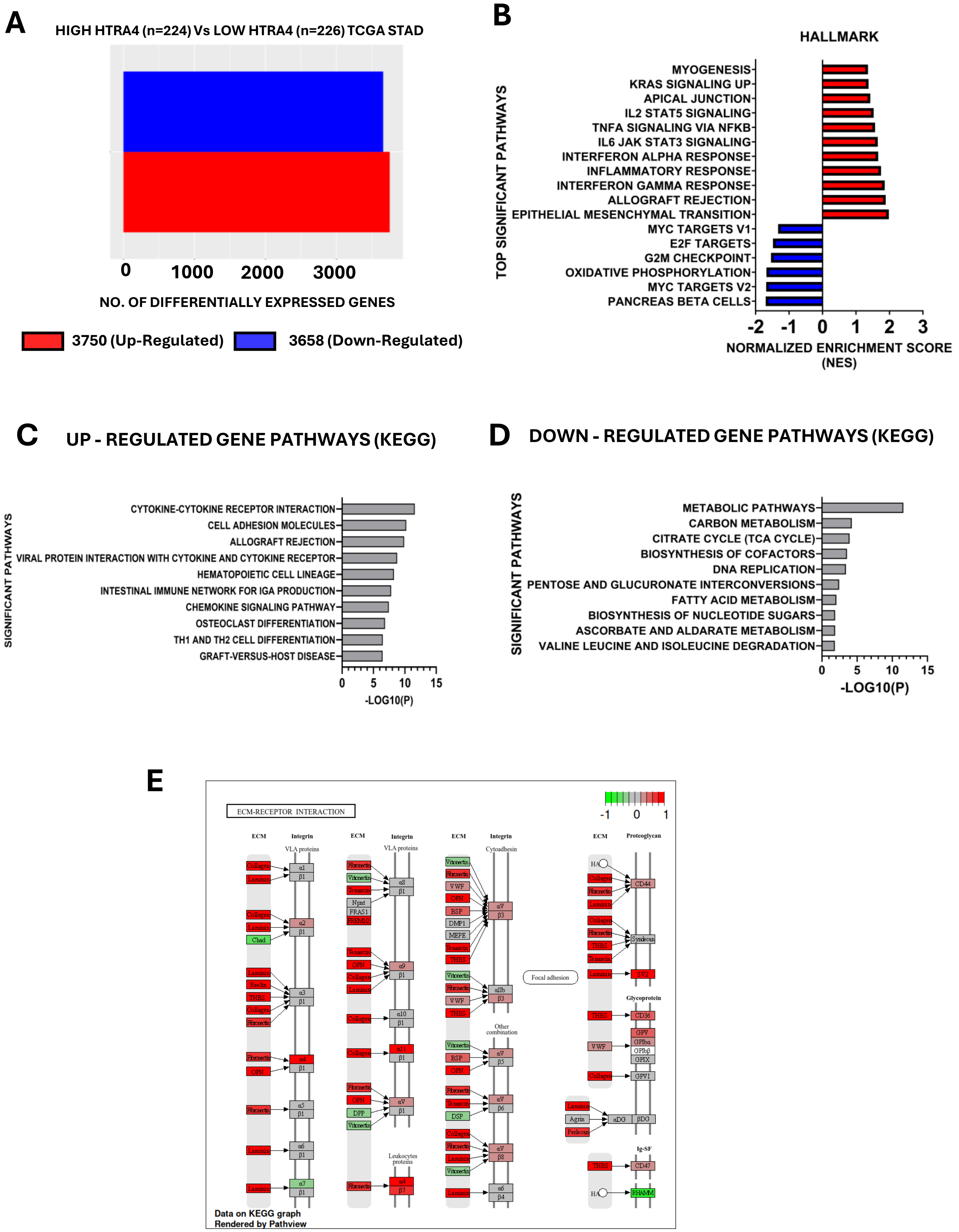

Supplementary Figure 12

CELL MARKERS FOR ANGIOGENESIS AND TUBULATION

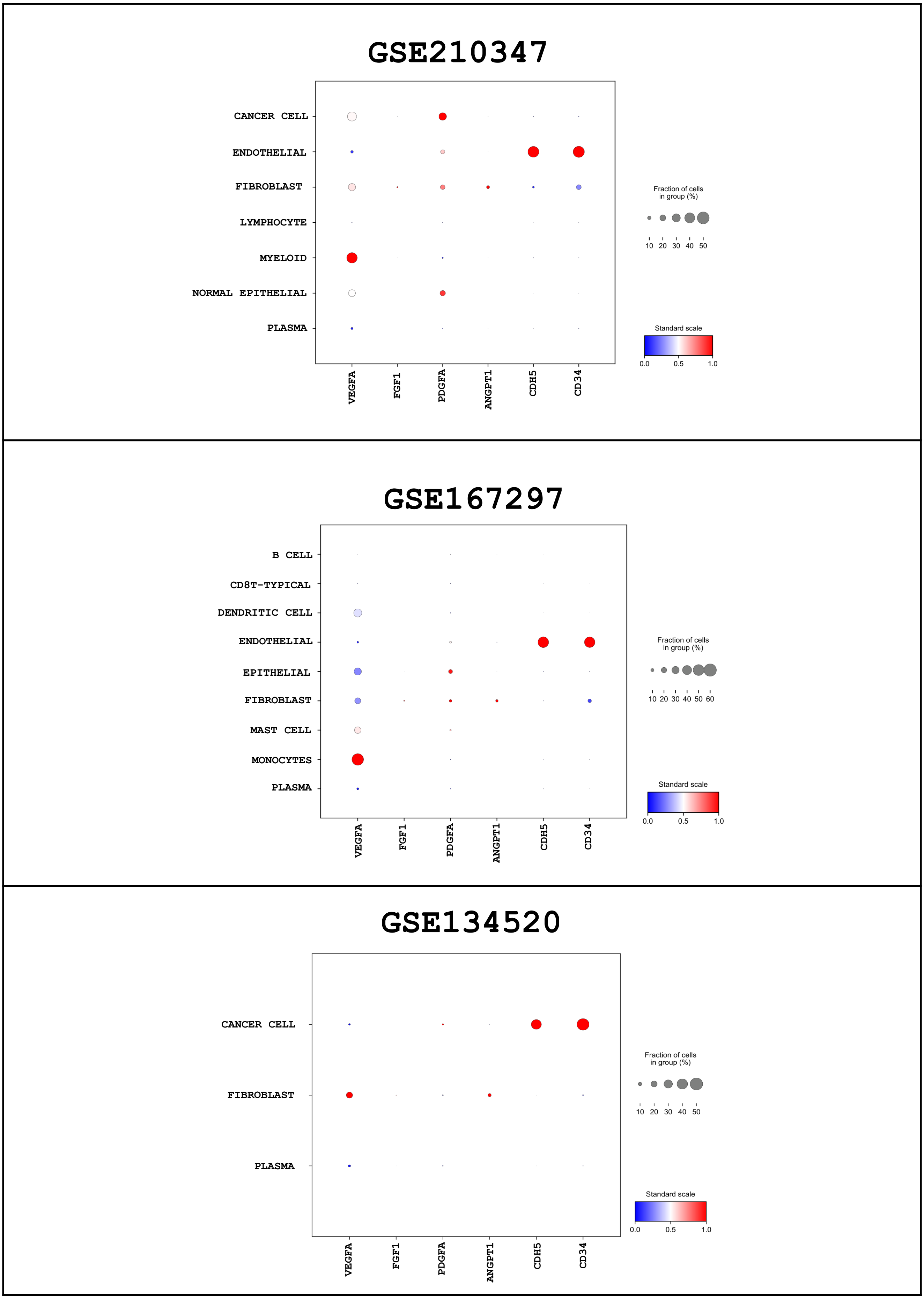

Supplementary Figure 13

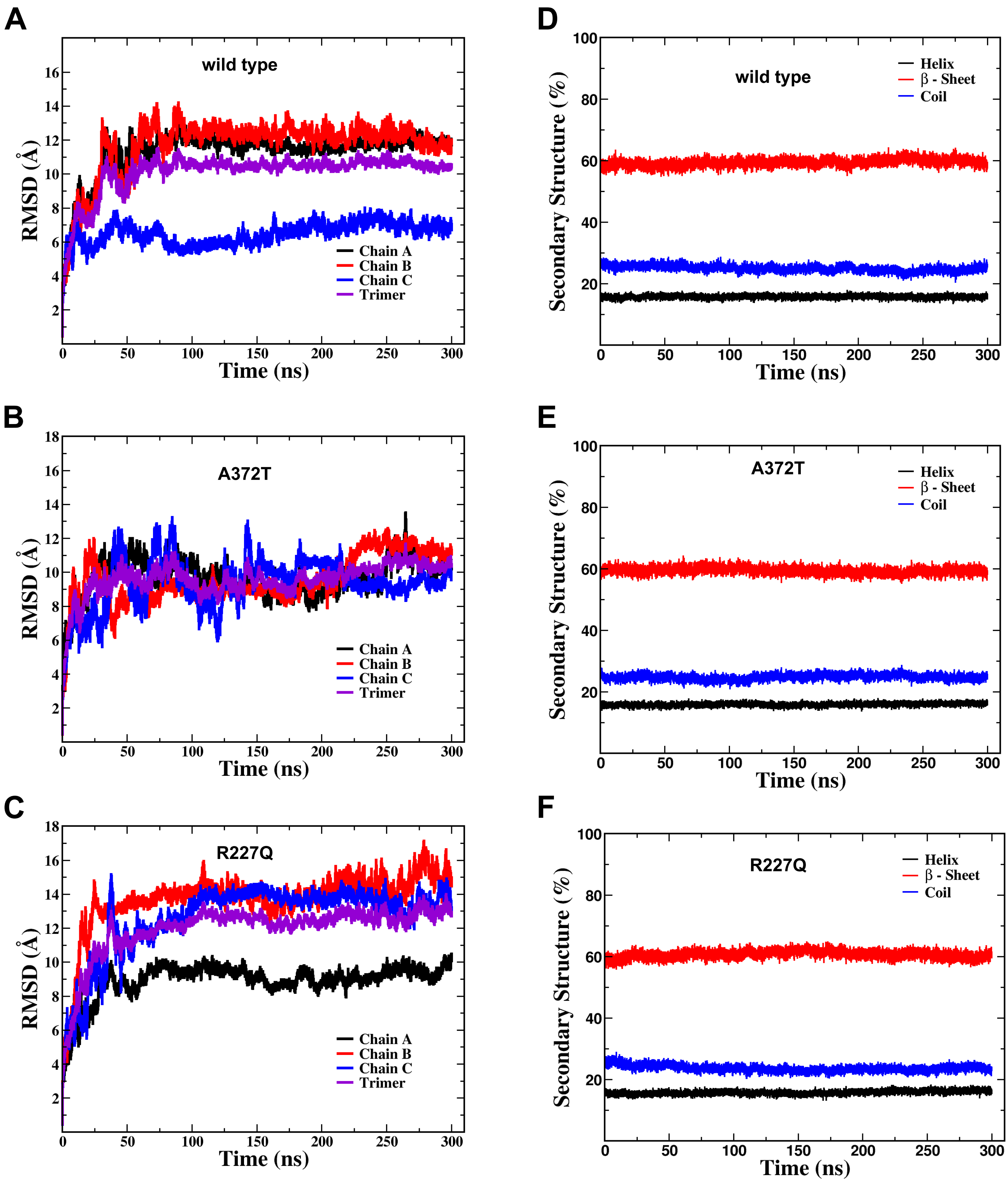

Supplementary Figure 14

A

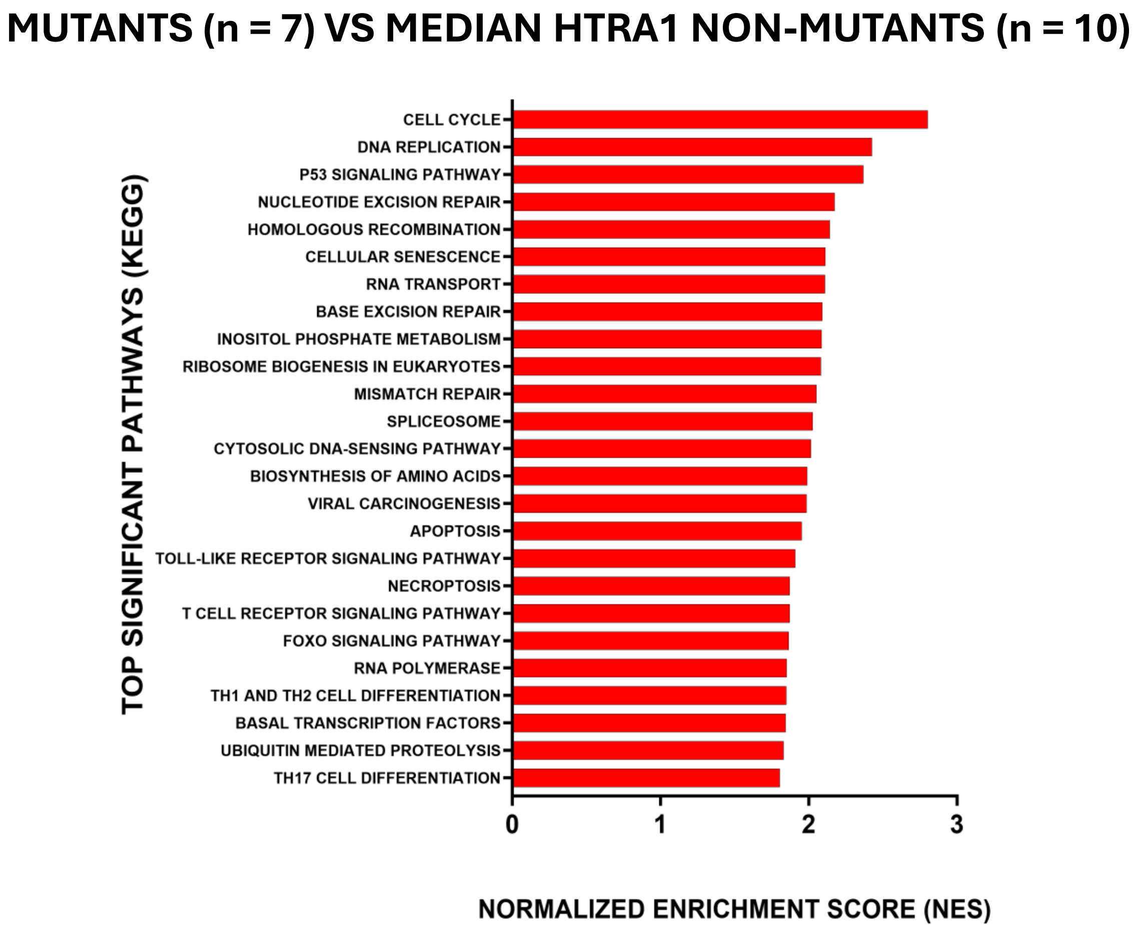

B

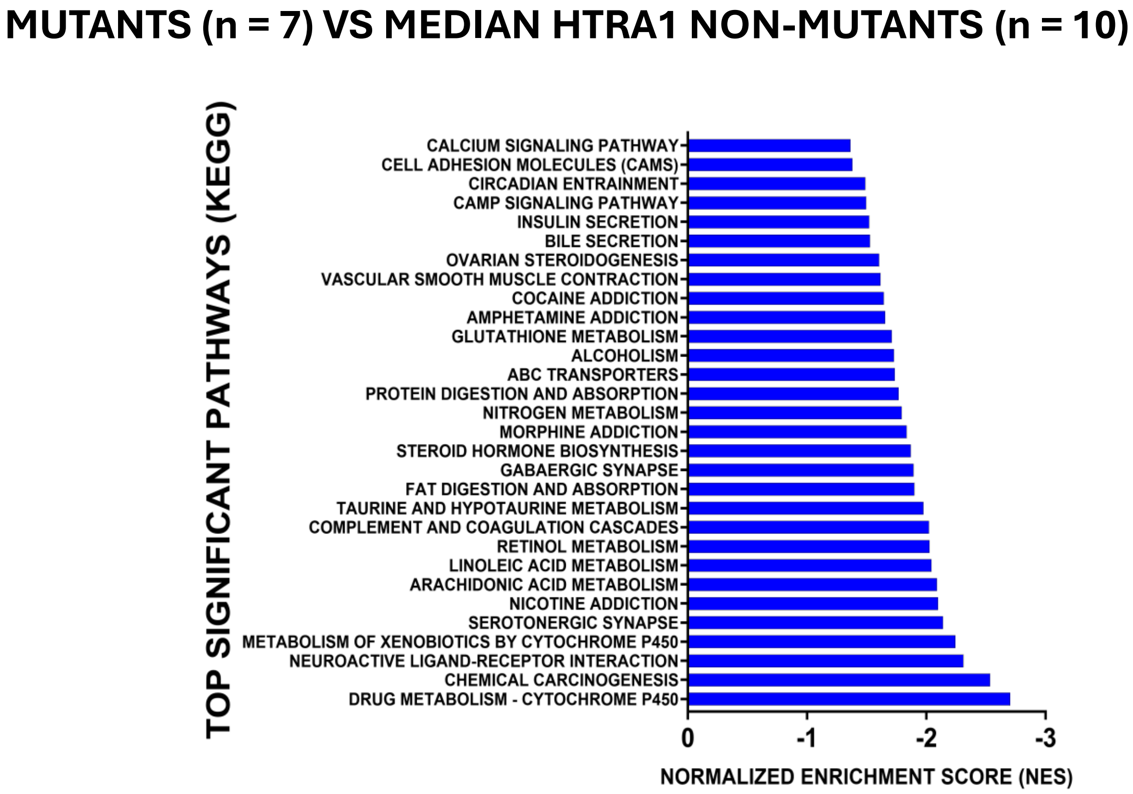

Supplementary Figure 15
